# Beyond width and density: stable carbon and oxygen isotopes in cork-rings provide insights of physiological responses to water stress in *Quercus suber* L

**DOI:** 10.7717/peerj.14270

**Published:** 2022-11-14

**Authors:** Augusta Costa, Paolo Cherubini, José Graça, Heinrich Spiecker, Inês Barbosa, Cristina Máguas

**Affiliations:** 1 Instituto Nacional de Investigação Agrária e Veterinária, I.P., Oeiras, Portugal; 2 Center for Environmental and Sustainability Research, NOVA University of Lisbon, Caparica, Portugal, Caparica, Portugal; 3 Swiss Federal Research Institute WSL, Birmensdorf, Switzerland; 4 Department of Forest and Nature Conservation, Faculty of Forestry, Technical University of British Columbia, Vancouver BC, Canada; 5 Instituto Superior de Agronomia, Centro de Estudos Florestais, Universidade de Lisboa, Lisboa, Portugal; 6 Chair of Forest Growth and Dendroecology, Albert-Ludwigs-University, Freiburg, Germany; 7 Faculdade de Ciências—cE3c, Centre for Ecology, Evolution and Environmental Changes, Universidade de Lisboa, Lisboa, Portugal

**Keywords:** Mediterranean forests, Cork oak, Stable isotopes, Climate change, Drought, Cork growth

## Abstract

As climate change increasingly affects forest ecosystems, detailed understanding of major effects is important to anticipate their consequences under future climate scenarios. The Mediterranean region is a prominent climate change hotspot, and evergreen cork oak (*Quercus suber* L.) woodlands are particularly climatically sensitive due to cork (bark) harvesting. Cork oak’s drought avoidance strategy is well-known and includes structural and physiological adaptations that maximise soil water uptake and transport and limit water use, potentially leading to reduced stem and cork growth. Trees’ responses to cope with water-limited conditions have been extensively described based on cork-rings width and, more recently, on cork-rings density, in dendroecological studies. However, so far, tree functional attributes and physiological strategies, namely photosynthetic metabolism adjustments affecting cork formation, have never been addressed and/or integrated on these previous cork-rings-based studies. In this study, we address the relation between carbon and oxygen stable isotopes of cork rings and precipitation and temperature, in two distinct locations of southwestern Portugal–the (wetter) Tagus basin peneplain and the (drier) Grândola mountains. We aimed at assessing whether the two climatic factors affect cork-ring isotopic composition under contrasting conditions of water availability, and, therefore, if carbon and oxygen signatures in cork can reflect tree functional (physiological and structural) responses to stressful conditions, which might be aggravated by climate change. Our results indicate differences between the study areas. At the drier site, the stronger statistically significant negative cork *δ*^13^C correlations were found with mean temperature, whereas strong positive cork *δ*^18^O correlations were fewer and found only with precipitation. Moreover, at the wetter site, cork rings are enriched in ^18^O and depleted in ^13^C, indicating, respectively, shallow groundwater as the water source for physiological processes related with biosynthesis of non-photosynthetic secondary tissues, such as suberin, and a weak stomatal regulation under high water availability, consistent with non-existent water availability constrains. In contrast, at the drier site, trees use water from deeper ground layers, depleted in ^18^O, and strongly regulate stomatal conductance under water stress, thus reducing photosynthetic carbon uptake and probably relying on stored carbon reserves for cork ring formation. These results suggest that although stable isotopes signatures in cork rings are not proxies for net growth, they may be (fairly) robust indicators of trees’ physiological and structural adjustments to climate and environmental changes in Mediterranean environments.

## Introduction

Evergreen cork oak (*Quercus suber* L.) forests cover about 2.2 million hectares in the western Mediterranean basin region (EUFORGEN, 2019) and cork is the sixth-most important non-wood forest product globally (FAO, 2014). Located in one of the most vulnerable regions of the globe (IPCC , 2017), these forests are currently experiencing a great impact on their growth and cork yield (Mendes et al., 2019; Costa & Cherubini, 2021) driven by climate change, lower precipitation, increased summer temperatures and more frequent and severe droughts (Garzón, Dios & Ollero, 2008; Cherubini, Battipaglia & Innes, 2021).

Cork oak produces narrow and ill-defined wood rings, contrasting with wide and sharp cork rings that are enhanced by cork harvesting (Pereira, 2007). So far, numerous dendroecological studies based on the width (Caritat, Gutiérrez & Molinas, 2000; Costa et al., 2015; Oliveira, Lauw & Pereira, 2016; Ghalem et al., 2018) and, more recently, on the chemical composition (Leite et al., 2020) and on the density of cork rings (Costa et al., 2022), have contributed to improve our knowledge on cork growth responses to inter-annual climate variability. However, despite this increased understanding of trees (and mainly cork) growth responses, these have scarcely been integrated with information on tree responses to water stress and/or under adverse environmental conditions. Only recently, the species’ drought-avoidance strategy to cope with summer droughts (Kurz-Besson et al., 2006) was reported to affect cork growth responses, exacerbated by the pressure of the cork harvesting and under stressful environmental conditions (Costa et al., 2016; Mendes et al., 2016).

Stable isotopes in tree rings are widely applied in dendroecological studies to better understand tree physiological responses to environmental changes in temperate regions (Lebourgeois, 2000; Ferrio et al., 2003; Saurer et al., 2008; Altieri et al., 2015). Although stable isotopes do not indicate net growth, they reflect tree functional attributes and physiological processes, namely photosynthetic metabolism adjustments, to cope with climate variations, under specific environmental conditions (Loader et al., 2008). Apart from differences and changes in the stable isotopic composition of different potential sources (*e.g.*,  *δ*^13^C of atmospheric CO_2_, for carbon or *δ*^18^O of water sources, for oxygen), the ratios of stable carbon and oxygen isotopes in tree rings are valuable to retrieve information on tree’s intrinsic water-use efficiency (*δ*^13^C) and soil water sources (*δ*^18^O) (O’Leary, 1988; Araguás-Araguás, Froehlich & Rozanski, 2000; Scheidegger et al., 2000; Loader, Robertson & McCarroll, 2003; McCarroll & Loader, 2004; Kurz-Besson et al., 2006; Ferrio et al., 2015; Hafner et al., 2015).

Extending stable isotope analysis to cork rings appears a very attractive way to go beyond variability in net cork (tree) growth (Oliveira, Lauw & Pereira, 2016; Ghalem et al., 2018; Leite et al., 2020; Costa & Cherubini, 2021; Costa et al., 2022) and to address, in an integrative way, the physiological and photosynthetic metabolism adaptation of cork oak to climate changes. To our knowledge, such an approach has not yet been made and no studies on the stable isotopic composition of cork are available. We aimed to address this issue and postulated that, similarly to tree-ring-based (using wood or cellulose) studies, cork-ring-based (using cork or suberin) dendroecological assessments through stable isotope ratios would indicate differential adaptations of trees to local water-limited environmental conditions under Mediterranean climate. Suberin is the most important structural component of cork cell walls (Graça & Pereira, 2000) and its role is thus comparable to cellulose in wood cell walls. However, suberin is fundamentally different from cellulose (or lignin) as it is a lipid-based polymer (Graça, 2015) and, therefore, distinct biochemical effects during cellulose and suberin synthesis might occur affecting its carbon and oxygen isotope ratios, both still unknown in cork oak.

In our exploratory study on cork rings, we hypothesize that in contrasting soil water availability environments, the variation of *δ*^13^C in the non-photosynthetic tissues such as cork reflects tree adjustments in stomatal conductance and a biochemical impairment of photosynthetic rates due to increased water scarcity during the hot and dry summer. In turn, the variation of cork-ring *δ*^18^O ratios would indicate trees physiological adaptation to environmental conditions, enabling the use of distinct water sources in the pathways of secondary tissues metabolism leading to suberin (and cork) formation (Barbour, Walcroft & Farquhar, 2002; Barbour, 2007; Sternberg & Ellsworth, 2011; Song, Clark & Helliker, 2014). To test these hypotheses, we analyzed cork samples from two locations where trees have potentially divergent and site-specific strategies to cope with summer drought: a peneplain site, where trees are able to reach shallow groundwater during summer (Mendes et al., 2016) and grown continuously, with no summer rest period (Costa, Pereira & Oliveira, 2002), and a hilly site where summer water stress leads to a drought-imposed rest, and trees eventually stop growing in a drought-avoidance strategy (Cherubini et al., 2003; Costa et al., 2022).

## Survey Methodology

### Study areas

**Figure 1:**
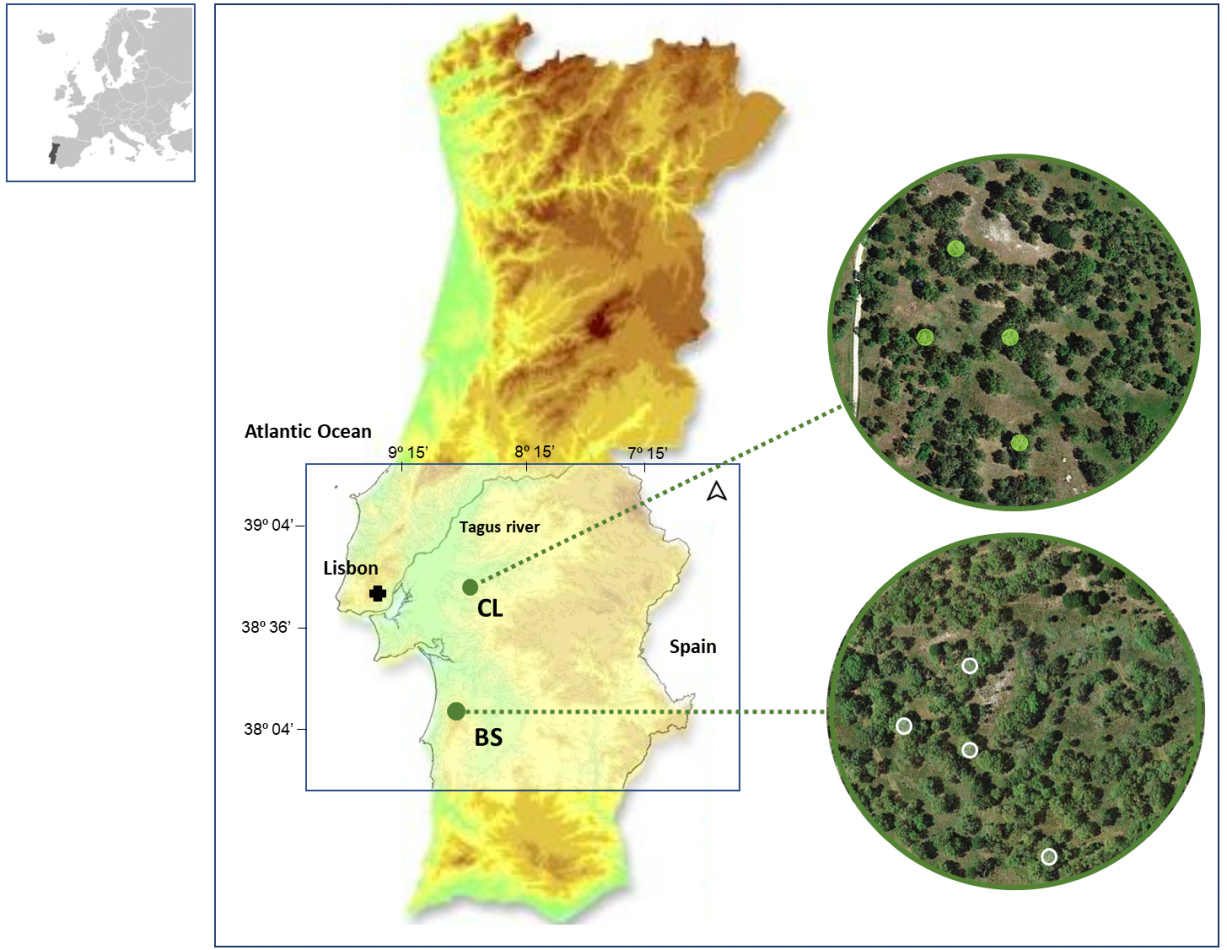
Location of the two study areas in SW Portugal, at Benavente (CL) and Grândola (BS). Colored Contour areas indicate elevation classes, at gaps of 50 m, between 10 m a.s.l. (*e.g.*, in CL, in green) and over 250 m a.s.l. (*e.g.*, in BS, in light brown). In the image composition, aerial images are showing both cork oak woodlands with the sampled trees highlighted.

In this study, we collected data from cork rings of selected trees in two distinct cork oak woodlands located in southwestern Portugal: Benavente (CL) (38°49′N, 8°49′W, 20 m a.s.l.) and ; Grândola (BS) (38°11′N, 8°37′W, 270 m a.s.l.) (Fig. 1). These cork oak woodlands have been described by the authors in their previous works (Costa et al., 2022; Costa et al., 2016).

Soil type, geologic formations and related biophysical site characteristics of these areas (CL and BS), fully described in previous work (Costa, Pereira & Oliveira, 2002; Costa et al., 2016; Costa et al., 2020; Mendes & Ribeiro, 2010; Mendes et al., 2016; Mendes et al., 2019), have distinctly affected soil water availability, specifically during the dry and hot summer season. At CL, trees have access to shallow groundwater, the main reliable water source for the maintenance of their physiologic activity during summer (Costa, Pereira & Oliveira, 2002). In contrast, trees at BS have a summer dormancy period (Oliveira et al., 1994) that is a drought stress-imposed rest period (Cherubini et al., 2003). At BS, tree growth must rely on groundwater that it is only used when the water table is reachable by tree roots, in early spring (Costa, Madeira & Oliveira, 2008; Costa et al., 2016).

Both study areas have a Mediterranean climate characterized by dry and hot summers and rainy winters fully described in Costa et al. (2022).

Precipitation and temperature raw data were obtained from the SNIRH (*Sistema Nacional de Informação de Recursos Hídricos*) database and were computed to obtain series of climate parameters for the period 1962-2012. Precipitation and temperature were calculated monthly, from February of the previous year to November of the current growth year. One (monthly), five- and twelve-months response functions were established for cork-rings *δ*^13^C, *δ*^18^O, width (C_RW_) and maximum density (C_MD_).

### Cork ring *δ*^13^C and *δ*^18^O data collection

**Figure 2:**
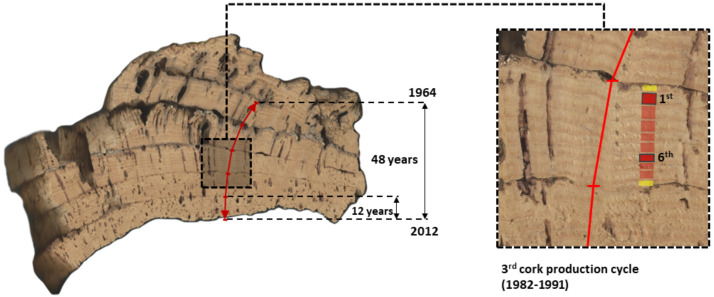
Image of a cross-section of one multi-layered cork sample from CL (Benavente) showing the five consecutive cork production cycles that were used in this study to extract and measure long-term series of reproduction cork rings (1964–2012). This cork sample (c. 15–30 cm) was collected in the tree stem at the (maximum cork harvesting) height of 1.80 m above ground. The considered five cork production cycles (1st to 5th, between 1964 and 2012) are identified and clearly visible, each encompassing 9 years, except for the most recent one, with 12 years (2000–2012). In the image composition, the third cork production cycle (1982–1991) was amplified showing their cork-rings with the first and sixth cork-rings highlighted.

Cork samples were collected from four healthy trees at each site (CL and BS) during the cork harvesting seasons (June) of 2012 and 2013. Such cork samples are complex, multi-layered samples, encompassing reproduction cork-rings that were induced by multiple and consecutive cork harvests (Fig. 2). Thus, these samples allow addressing long-term chronologies of cork rings in individual trees (Costa et al., 2015; Costa et al., 2022). In each sample, cork rings were visually dated using image analysis techniques (Costa et al., 2015; Costa & Cherubini, 2021) for the period 1962–2013.

Generally, cork production cycles at the mountain site (BS) are longer (10 years) than at the peneplain site (CL) (9 years) (Costa et al., 2022). At BS, cork samples were harvested in 2012, all comprised five consecutive cork production cycles of 10 years, initiated in 1962 (first cork production cycle), 1972, 1982, 1992 and 2002 (fifth cork production cycle), with the final cork harvest in 2012. At CL, cork samples per tree were harvested in 2013, to which corresponded five consecutive cork production cycles of 9 years starting in 1968 (first), 1977, 1986, 1995 and 2004 (fifth), and harvested in 2012 to which corresponded five cork production cycles initiated in 1964 (first), 1973, 1982, 1991 and 2000 (exceptionally, the fifth cycle lasted 12 years) (Fig. 2).

Cork-ring width (C_RW_) and maximum density (C_MD_) chronologies were generated for each cork sample (Costa et al., 2022). Since C_RW_ is heavily influenced by cork harvesting, each cork-ring width series was detrended by fitting a polynomial function (Costa, Pereira & Oliveira, 2002). Cork-ring width indices (residuals) (IC_RW_) were calculated by dividing the observed C_RW_ values by the predicted ones and IC_RW_ chronologies were then built. On the other hand, C_MD_ chronologies did not seem to contain any cork harvesting related trend (Costa et al., 2022) and therefore did not require any statistical detrending. Cork-ring maximum density indices (IC_MD_) were calculated by dividing the observed C_MD_ values by their mean value in each cork production cycle and, this way the IC_MD_ chronologies were built.

Due to technical constrains to obtain enough cork mass for isotopic analysis, not all cork rings could be analyzed. In all samples, we selected the 1st and the 6th rings, respectively with the cork age (*y*_cork_) equal to 1 and 6, of each of the considered five production cycles. The 1st ring is the largest one, representative of the trees response to cork harvesting and the 6th ring is a middle cycle ring, representative of an average cork growth, in the absence of stressful disturbances (Fig. 2). Data on carbon and oxygen isotopes were thus ascribed to both cork ages in all the consecutive cycles, regardless of the time series. By selecting fixed years (1st and 6th cork rings) of each cork-production cycle we expected to obtain information reflecting the intra- and inter-tree variability in tree physiological responses to drought conditions, under the influence of cork harvesting. A total of 78-records for (*δ*^13^C) and for (*δ*^18^O) were collected in the cork-rings of the study areas, 44 records for BS and 34 for CL.

Stable isotope ratio analyses were performed at the Stable Isotopes and Instrumental Analysis Facility (SIIAF) of the Centre for Ecology, Evolution and Environmental Changes (cE3c), University of Lisbon, Portugal. The stable-carbon isotope ratio (^13^C/^12^C) of cork was measured by continuous flow isotope mass spectrometry (CF-IRMS), on a Sercon Hydra 20-22 (Sercon, Hook, Hampshire, UK) stable isotope ratio mass spectrometer, coupled to a EuroEA (EuroVector, Italy) elemental analyzer for online sample preparation by Dumas-combustion. The stable-oxygen isotope ratio (^18^O/^16^O) of cork and suberin was also determined by continuous flow isotope mass spectrometry (CF-IRMS), coupled to a high temperature pyrolysis unit (HT-PyrOH, Eurovector, Italy) and a EuroEA3000 (EuroVector, Lombardy, Italy) elemental analyzer for online sample preparation by thermal degradation (thermolysis). The precision of the isotope ratio analysis, calculated using values from six to nine replicates of standard material interspersed among samples in every batch analysis, was ≤0.2‰. Results are given in the *δ*-notation, *i.e.,* in the delta notation relative to the international standards:

\begin{eqnarray*}\delta \mathrm{sample}=(\mathrm{Rsample}/\mathrm{Rstandard}-1)\times \text{1,000}, \end{eqnarray*}

where Rsample is the molar fraction of ^13^C/^12^C, or ^18^O/^16^O ratio of the sample and Rstandard, of the standards of Vienna Pee Dee Belemnite (VPDB) for carbon and Vienna Standard Mean Ocean Water (VSMOW) for oxygen.

### Statistical analyses

The climate-isotope relationships were tested based on monthly Pearson correlations considering one month and five- and twelve-months windows, from February of the prior growth year till November of the current growth year. We included prior-year climate variables in the analysis because climate in the preceding growing season often influences stable isotope values in the following season due to carbon carry-over effects (Gessler et al., 2014).

Linear mixed-effects modelling was used to examine the influence of the covariate cork age (*y*_cork_) at the two sites (non-water-limited area, CL and water-limited area, BS) on the isotope-derived physiological responses of cork oak and; to examine the variability of the cork *δ*^13^C and *δ*^18^O relationships with climate parameters, precipitation and temperature. Climate variables were thus considered as the fixed factors for the response functions *δ*^13^C and *δ*^18^O composition of cork rings. Also, linear mixed-effects models would explicitly integrate among-tree variability, considering trees as a random effect, related with tree responses to limited water availability. Model fitting was done by adding random effects of site *Xy*_cork_ (four classes) and tree (8 classes), through random intercept alone. We evaluated model performance using the Akaike information criterion (AIC). The model with the lowest AIC value was considered the best to predict response variables (*δ*^13^C and *δ*^18^O). We further evaluated the variation explained by the random and fixed effects together by calculating marginal R^2^ to define the error or statistical uncertainty related with the inter-tree variability. All the analyses were conducted in R statistical environment (R Development Core Team, 2019) using the *nlme* package in R.

## Results

### *δ*^13^C composition in cork rings and its correlations with climate

**Figure 3:**
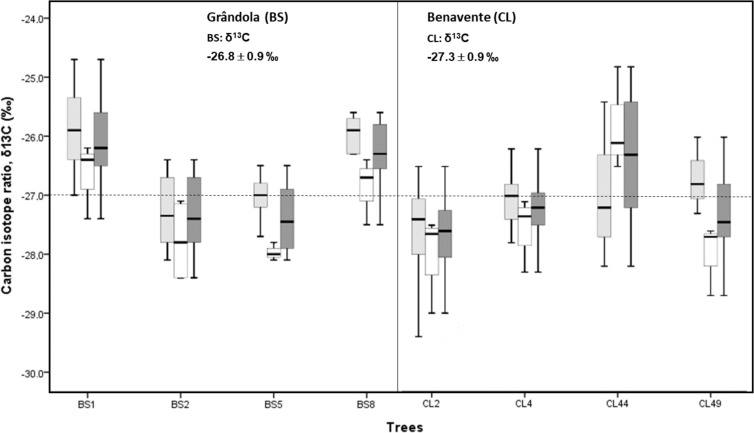
Variability of *δ*^13^C in cork-rings (*n* = 78) of the four trees from the two study areas, Grândola (*n* = 44) (BS) (in the left) and Benavente (*n* = 34) (CL) (in the right). Solid light grey boxes for *y*_cork_ = 1 (1st cork growth years) (*n* = 24 for BS and *n* = 22 for CL); solid white boxes for *y*_cork_ = 2 (6th cork growth years) (*n* = 20 for BS and *n* = 12 for CL) and solid dark grey boxes for all cork growth years. Multi-boxplots (median; box, interquartile range: IQR = Q75percent − Q25percent; whiskers, minimum and maximum). For each study area, mean ± standard deviation of *δ*^13^C in cork-rings. Dotted line is for average value of cork *δ*^13^C of −27.0 ‰.

The mean isotopic compositions (*δ*^13^C) of corkrings ranged between −27.3‰ at CL and −26.8‰ at BS (Fig. 3). Average *δ*^13^C values at BS indicated that here cork was slightly more enriched in ^13^C (over 0.5‰) than at CL. However, there was no significant difference in cork-rings *δ*^13^C between the trees from BS and CL (nested ANOVA results showed no significant differences between the two groups (sites), *F*(1, 6) = 0.676, *p*-value = 0.442). Between trees within each study area, the variations were statistically significant (nested ANOVA results showed *F*(6, 70) = 6.951, *p*-value = 0.000). At CL, the cork of tree CL44 differed significantly from the other trees and; at BS, the cork of trees BS1 and BS8 differed significantly from the other two trees. CL44, BS1 and B8 were distinctly enriched in ^13^C (less negative *δ*^13^C values) when compared to the other trees (Fig. 3).

Furthermore, in all cork samples, except for the cork of tree CL44 (at CL), the mean isotopic compositions (*δ*^13^C) of 1st cork-rings (*y*_cork_ = 1) were less negative than 6th cork-rings (*y*_cork_ = 6) which indicates that, on average, cork formed immediately after cork harvesting is more enriched in ^13^C when compared to the cork formed later, hereafter the 6th year in the cork harvesting cycle.

**Figure 4:**
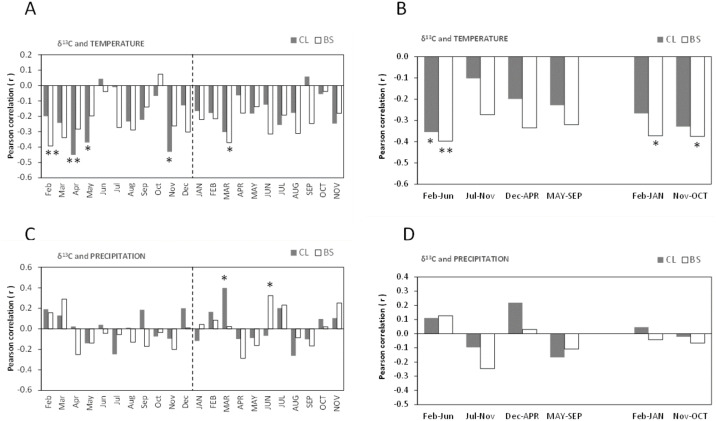
Pearson correlation coefficient (*r*) between *δ*^13^C of all cork rings from Grândola (*n* = 44) (BS) and Benavente (*n* = 34) (CL) and: (A) Monthly mean temperature from February previous to the current growth year (Feb) to November of current growth year (NOV). Significant correlations were found for BS in: Feb (*r* =  − 0.393**, *p*-value = 0.008) and MAR (*r* =  − 0.371*, *p*-value = 0.013) and, for CL in: Apr (*r* =  − 0.451**, *p*-value = 0.008); May (*r* =  − 0.370*, *p*-value = 0.031) and in Nov (*r* =  − 0.431*, *p*-value = 0.011). (B) Selected five- and twelve-months mean temperature. Significant correlations were found for BS in: Feb–Jun (*r* =  − 0.397**, *p*-value = 0.008); in Feb–JAN (*r* =  − 0.372*, *p*-value = 0.013) and in Nov–OCT (*r* =  − 0.375*, *p*-value = 0.013) and, for CL in: Feb–Jun (*r* =  − 0.355*, *p*-value = 0.039). (C) Monthly precipitation. Significant correlations were found for BS in: JUN (*r* = 0.324*, *p*-value = 0.032) and for CL in MAR (*r* = 0.398*, *p*-value = 0.020); (D) Selected five- and twelve-months precipitation. Asterisks represent significant correlations (*, *p*-value < 0.05 and **, *p*-value < 0.01). Lower case, months of year prior to the growth year; capitals, months of current growth year.

The Pearson’s correlations (r) between *δ*^13^C of cork rings and mean temperature showed similarities among the trees of CL and BS, and were significantly negative (Figs. 4A–4B). This is a striking result which suggests that the *δ*^13^C (‰) of cork rings will be lower in warmer years, *i.e.,* cork will be more ^13^C depleted.

Noticeably, besides a few significant monthly correlations (Fig. 4A), the relationships became more robust after grouping temperature variables in periods of 5- or 12-months, mainly at BS (Fig. 4B). The variation of cork *δ*^13^C at BS was much more sensitive to temperature than at CL, with a marked sensitivity to prior conditions since spring of the previous year throughout the growth year (Fig. 4B). The highest negative correlation coefficients were found for the mean temperatures previous to the growth year, in February–September (*r* =  − 0.423, *p*-value = 0.004) at BS and in April–May (*r* =  − 0.462, *p*-value = 0.006) at CL. Moreover, at BS, *δ*^13^C also correlated with temperature of the current growing season, in the spring period (March = −0.371, *p*-value = 0.013) (Figs. 4A–4B), in the onset of the cork growth flush.

Weaker correlations were found between *δ*^13^C of cork rings and precipitation in both study areas (Figs. 4C–4D). The precipitation of current-year March (at CL) and June (at BS) positively influenced cork *δ*^13^C (Fig. 4C). In contrast with temperature, no significant correlations were found with grouped precipitation values (periods of 5- to 12-months) (Fig. 4D). A relatively weak correlation was observed only at BS, in the summer of the current growth year in June–August (*r* = 0.350; *p*-value = 0.021). Also, in contrast with temperature, results revealed one irregularity of the relationship between cork *δ*^13^C and precipitation: although not statistically significant, correlations were either positive or negative correlations, regardless of the considered periods (Fig. 4D).

### *δ*^18^O composition in cork rings and its correlations with climate

**Figure 5:**
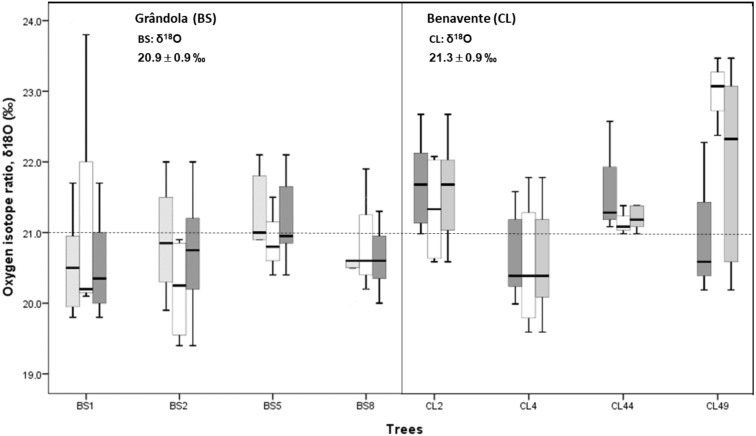
Variability of cork stable isotope ratios, *δ*^18^O in cork-rings (*n* = 78) of the four trees from the two study areas, Grândola (*n* = 44) (BS) (in the left) and Benavente (*n* = 34) (CL) (in the right). Solid light grey boxes for *y*_cork_ = 1 (1st cork growth years) (*n* = 24 for BS and *n* = 22 for CL); Solid withe boxes for *y*_cork_ = 2 (6th cork growth years) (*n* = 20 for BS and *n* = 12 for CL) and solid dark grey boxes for all cork growth years. Multi-boxplots (median; box, interquartile range: IQR = Q75percent − Q25percent; whiskers, minimum and maximum). For each study area, mean ± standard deviation of *δ*^18^O in cork-rings. Dotted line is for average value of cork *δ*^18^O of 21.0 ‰.

Cork-ring *δ*^18^O averaged 21.3 ± 0.9‰ (CL) and 20.9 ± 0.9‰ (BS). Average *δ*^18^O values indicate that at CL, cork was enriched in ^18^O, over 0.4‰ more than at BS (Fig. 5). However, there was no significant difference in cork-rings *δ*^18^O between the trees from BS and CL (nested ANOVA results showed no significant differences between the two groups (sites), *F*(1, 6) = 3.188, *p*-value = 0.121). Between trees within each study area, the variations were not statistically significant (nested ANOVA results showed *F*(6, 70) = 1.989, *p*-value = 0.079). Furthermore, mean values of suberin’s *δ*^18^O in cork rings ranged between 16.8‰ at CL and 16.3‰ at BS, in accordance with the values found for cork.

At CL, the cork of tree CL4 differed significantly from all other trees, and it was depleted in ^18^O when compared to the other trees (Fig. 5).

**Figure 6:**
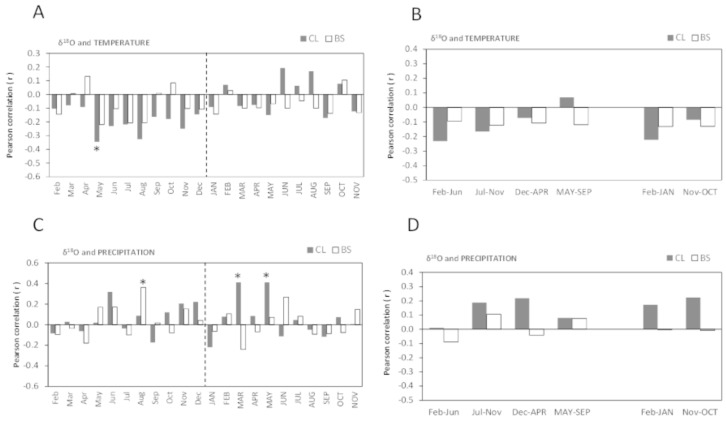
Pearson correlation coefficient (*r*) between *δ*^18^O of all cork rings from Grândola (*n* = 44) (BS) and Benavente (*n* = 34) (CL) and: (A) Monthly mean temperature from February previous to the current growth year (Feb) to November of current growth year (NOV). Significant correlations were found for CL in: May (*r* =  − 0.345*, *p*-value = 0.046); (B) Selected five- and twelve-months mean temperature; (C) Monthly precipitation. Significant correlations were found for BS in: Aug (*r* = 0.362*, *p* = 0.016) and for CL in MAR (*r* = 0.411*, *p*-value = 0.016) and in MAY (*r* = 0.411*, *p*-value = 0.016); (D) Selected five- and twelve-months precipitation. Asterisks represent significant correlations (*, *p*-value < 0.05). Lower case, months of year prior to the growth year; capitals, months of current growth year.

In all cork samples, except for the cork of tree CL49 (at CL), the mean isotopic compositions (*δ*^18^O) of 1st cork-rings (*y*_cork_ = 1) were slightly higher or equal than 6th cork-rings (*y*_cork_ = 6) which indicates that cork formed immediately after cork harvesting is somewhat more enriched in ^18^O when compared to the cork formed later, in the 6th year after the cork harvesting (Fig. 6).

The oxygen isotope signal in cork rings is more sensitive to precipitation than to temperature (Fig. 6). The correlations between *δ*^18^O and mean temperature were statistically significant only in trees at CL (Figs. 6A–6B). At this site, we found negative correlations with the temperature only in spring prior to the growth year. Besides the weak isolated monthly correlation with May (*r* =  − 0.345, *p*-value = 0.046) (Fig. 6A), this relationship became slightly more robust when after grouping temperature shows statistically significant correlations, in the summer prior to the growth year, between May–June (*r* =  − 0.373, *p*-value = 0.030) and May–August (*r* =  − 0.359, *p*-value = 0.037).

The correlations found between *δ*^18^O and precipitation were weak and negligible at BS but not at CL (Figs. 6C–6D). Here, *δ*^18^O relationship was positive and statistically significant with precipitation in the current spring, in March–May (*r* = 0.497, *p*-value = 0.003) and in spring-early summer, in March–June (*r* = 0.428, *p*-value = 0.012).

### Correlations between width and density of cork-rings and climate

At the two study areas, none of the correlations between cork-ring isotopic compositions (*δ*^13^C and *δ*^18^O), cork-ring width (IC_RW_) and cork-ring maximum density (IC_MD_) were statistically significant.

The two study areas clearly differ in the relation between IC_RW_ and climate parameters. At BS, the results are inconsistent regarding precipitation: (i) within the year prior to the growth year, two distinct sub-periods: one in the spring (March–June), positively correlated with precipitation, and another in summer-autumn (June–October), negatively correlated and; (ii) in the current growth year, contrasting weaker positive correlations in summer-autumn (July–September) (Table 1). Here, despite the non-significant relation between IC_RW_ and mean temperature of the current growth year (in line with the non-significant relation with IC_MD_, Table 1), the mean temperature within March and November prior to the growth year influenced cork growth much more than the cork density (Table 1).

**Table 1:** Selected significant correlation coefficients (*r*) between IC_RW_ and IC_MD_ and precipitation and mean temperature, of all cork rings from Benavente (*n* = 40) (CL) and Grândola (*n* = 52) (BS).

Parameter	Study area	Precipitation		Temperature	
		Months	*r*	Months	*r*
IC_RW_	CL	February_(−1)_	−0.331^*^	April_(−1)_	0.349^*^
				April_(−1)_–May_(−1)_	0.376^*^
				December_(−1)_–February	−0.409^**^
		July–November	−0.329^*^		
	BS	March_(−1)_–June_(−1)_	0.297^*^	March_(−1)_–September_(−1)_	0.277^*^
		May_(−1)_	0.307^*^	April_(−1)_–September_(−1)_	0.284^*^
		June_(−1)_–October_(−1)_	−0.348^*^	May_(−1)_–November_(−1)_	0.281^*^
		September_(−1)_–October_(−1)_	−0.342^*^	August_(−1)_–September_(−1)_	0.279^*^
		October_(−1)_	−0.323^*^		
		July–September	0.298^*^		
		September	0.286^*^		
		September–October	0.283^*^		
IC_MD_	CL		**n.s.**		**n.s.**
	BS	May_(−1)_–October_(−1)_	−0.275^*^	May_(−1)_	0.278^*^
		May	0.273^*^		
		August	0.329^*^		

**Notes.**

Significant levels according to Student’s test: * *p* value < 0.05; ** *p* value < 0.01.

(−1) indicate the months in the year prior to the growth year.

At CL, an irregular pattern of correlations between ICRW and climate parameters was observed: negative correlations between precipitation and IC_RW_, mainly in July–November of the current growth year, striking, strong and positive correlations between mean temperature in spring (April–May) of the year prior to the growth year, and negative correlation with mean temperature in the winter prior to the growth year (December–February) (Table 1).

Relationships between climate parameters (precipitation and temperature) and IC_MD_ were only relevant at BS; at CL the correlations were statistically not significant (Table 1). At former site, precipitation conditions prior to the growth year negatively affected IC_MD_, from May to October. Conversely, the IC_MD_ correlations with precipitation of current growth year May and August were statistically significant and positive. Moreover, between temperature and IC_MD_, only a relative weak significant correlation was found, with mean temperature of May prior to the growth year (*r* = 0.278, *p*-value = 0.046) (Table 1).

The correlations found between mean temperature and IC_RW_ and IC_MD_ at BS, and between precipitation and IC_RW_ and IC_MD_ at CL, were considerably weaker than the correlations found with *δ*^13^C (BS) and with *δ*^18^O (CL).

### Influence of cork harvesting and site on the relation between climate and cork- rings isotope composition (*δ*^13^C and *δ*^18^O)

Selected mixed-effects linear models (M0–M5) were plotted against the site *Xy*_cork_ and trees random-effects (Table 2). The fixed-effect variables were the climate variables which displayed the highest bootstrap correlation coefficients. For *δ*^13^C were considered the mean temperatures of: February_(−1)_–June_(−1)_ (M1) (*r* =  − 0.355, *p*-value = 0.039 for CL and *r* =  − 0.397, *p*-value = 0.008 for BS); April_(−1)_–May_(−1)_ (M2) (*r* =  − 0.462, *p*-value = 0.006 for CL and *r* =  − 0.303, *p*-value = 0.013 for BS) and; March (M3) (*r* =  − 0.302, *p*-value = 0.082 for CL and *r* =  − 0.371, *p*-value = 0.013 for BS). For *δ*^18^O were considered the precipitation of: August_(−1)_ (M4) (*r* = 0.087, *p*-value = 0.624 for CL and *r* = 0.362, *p*-value = 0.016 for BS) and; March–June (M5) (*r* = 0.428, *p*-value = 0.012 for CL and *r* =  − 0.220, *p*-value = 0.157 for BS).

**Table 2:** Coefficients, AIC values and R^2^ for linear mixed-effects models of isotope ratios composition (*δ*^13^C and *δ*^18^O) of cork rings, with selected climatic variables as fixed effect and with site *Xy*_cork_ and trees, as random factors.

Isotope composition (*δ*^13^C)							
Climate variables	MODEL	Model equation	*α*_0_	*α*_1_	*σ*_sitexycorkj_(*μ*_*j*_)	*σ*_Treej_(*μ*_*j*_)	*σ*_*ɛij*_
Temperature							
February_−1_–June_(−1)_ (**C**_LIM1_)	M_1_	*δ*^13^C_*ij*_ = *α*_0_ + *α*_1_C_LIM1ij_ + *μ*_sitexycorkj_ + *ɛ*_*ij*_	−22.420	−0.308^**^	0.044		0.713
AIC	206.06					
${R}_{\text{(fixed effects)}}^{2}$	0.12						
${R}_{\text{(total)}}^{2}$	0.17						
February_−1_–June_(−1)_ (**C**_LIM1_)	M_10_	*δ*^13^C_*ij*_ = *α*_0_ + *α*_1_C_LIM1ij_ + *μ*_Treej_ + *ɛ*_*ij*_	−22.208	−0.319^**^		0.274	0.467
AIC	184.94						
${R}_{\text{(fixed effects)}}^{2}$	0.13						
${R}_{\text{(total)}}^{2}$	0.45						
April_−1_–May_(−1)_ (**C**_LIM2_)	M_2_	*δ*^13^C_*ij*_ = *α*_0_ + *α*_1_C_LIM2ij_ + *μ*_sitexycorkj_ + *ɛ*_*ij*_	−22.850	−0.267^**^	0.065		0.696
AIC	205.08						
${R}_{\text{(fixed effects)}}^{2}$	0.12						
${R}_{\text{(total)}}^{2}$	0.20						
April_−1_–May_(−1)_ (**C**_LIM2_)	M_20_	*δ*^13^C_*ij*_ = *α*_0_ + *α*_1_C_LIM2ij_ + *μ*_Treej_ + *ɛ*_*ij*_	−22.320	−0.296^**^		0.308	0.438
AIC	181.12						
${R}_{\text{(fixed effects)}}^{2}$	0.15						
${R}_{\text{(total)}}^{2}$	0.50						
March (**C**_LIM3_)	M_3_	*δ*^13^C_*ij*_ = *α*_0_ + *α*_1_C_LIM3ij_ + *μ*_sitexycorkj_ + *ɛ*_*ij*_	−24.163	−0.232^**^	0.031		0.741
AIC	208.32						
${R}_{\text{(fixed effects)}}^{2}$	0.11						
${R}_{\text{(total)}}^{2}$	0.14						
March (**C**_LIM3_)	M_30_	*δ*^13^C_*ij*_ = *α*_0_ + *α*_1_C_LIM3ij_ + *μ*_Treej_ + *ɛ*_*ij*_	−23.880	−0.251^**^		0.284	0.472
AIC	185.95						
${R}_{\text{(fixed effects)}}^{2}$	0.13						
${R}_{\text{(total)}}^{2}$	0.45						
Isotope composition (*δ*^18^**O**)							
Climate variables	MODEL	Model equation	*α*_0_	*α*_1_	*σ*_sitexycorkj_(*μ*_*j*_)	*σ*_Treej_(*μ*_*j*_)	*σ*_*ɛij*_
Precipitation							
August_(−1)_ (**C**_LIM4_)	M_4_	*δ*^18^O_*ij*_ = *α*_0_ + *α*_1_C_LIM4ij_ + *μ*_sitexycorkj_ + *ɛ*_*ij*_	20.914	0.029^*^	0.002		0.876
AIC	218.99						
${R}_{\text{(fixed effects)}}^{2}$	0.06						
${R}_{\text{(total)}}^{2}$	0.06						
August_(−1)_ (**C**_LIM4_)	M_40_	*δ*^18^O_*ij*_ = *α*_0_ + *α*_1_C_LIM4ij_ + *μ*_Treej_ + *ɛ*_*ij*_	20.959	0.027^*^		0.073	0.807
AIC	217.63						
${R}_{\text{(fixed effects)}}^{2}$	0.05						
${R}_{\text{(total)}}^{2}$	0.13						
March–June (**C**_LIM5_)	M_5_	*δ*^18^O_*ij*_ = *α*_0_ + *α*_1_C_LIM5ij_ + *μ*_sitexycorkj_ + *ɛ*_*ij*_	20.960	0.001^*^	0.005		0.917
AIC	220.29						
${R}_{\text{(fixed effects)}}^{2}$	0.01						
${R}_{\text{(total)}}^{2}$	0.01						
March–June (**C**_LIM5_)	M_50_	*δ*^18^O_*ij*_ = *α*_0_ + *α*_1_C_LIM5ij_ + *μ*_Treej_ + *ɛ*_*ij*_	21.000	0.008^*^		0.027	0.835
AIC	218.32						
${R}_{\text{(fixed effects)}}^{2}$	0.01						
${R}_{\text{(total)}}^{2}$	0.10						

**Notes.**

Significance of predictors is indicated by: * (*p*-value < 0.05) and ** (*p*-value < 0.01).

In all the models, *δ*^13^C in cork rings decreased with mean temperature and confirmed our previous results that *δ*^13^C of cork rings was lower in warmer years, *i.e.,* ^13^C depleted (Figs. 4A–4B). In the best model for *δ*^13^C (M2—with the lowest AIC of 205.08 and explaining about 20% of the total variability), *δ*^13^C decreased on average about −0.267‰ per °C of monthly spring temperature (April_(−1)_–May_(−1)_) (Table 2). Despite the general negative correlation, in this model the predicted values of *δ*^13^C in the cork-rings formed immediately after cork harvesting (*y*_cork_ = 1) at a drier site (BS) were clearly higher (less negative *δ*^13^C), with an *α*_0_ = −22.482, than in the later cork-rings (*y*_cork_ = 6) or at the CL wetter site (with either *y*_cork_ of 1 or 6), with an *α*_0_ = −23.004, for the same negative slope (*α*_1_ = −0.267, *p* < 0.001).

Model M20 for *δ*^13^C, with trees as random effect, had the lowest AIC, 181.12 and explained about 50% of the total variation (Table 2). This suggests a most strong influence of between-tree variability on the variations of cork-rings *δ*^13^C with mean temperature, mainly when compared to model M2 that explained a non-negligible 20% of total variation and that the site *Xy*_cork_ random effect explained up to 15% of total variability.

In the models with *δ*^18^O as the response function, *δ*^18^O increased with the monthly precipitation, in accordance with previous results on significant correlations (Figs. 6C–6D). The models M4 (with an AIC of 218.99 and explaining 6% of the total variation) and M5 (with an AIC of 220.29 and explaining 1% of the total variation), poorly explained the total variation. Furthermore, no variation was explained by the site *Xy*_cork_ random effect (Table 2). However, in the random intercept model (M5), the predicted values of *δ*^18^O in cork-rings formed at a drier site (BS) were clearly different and slightly depleted in ^18^O (lower *δ*^18^O, with an *α*_0_ = 20.772), than in the wetter site (CL), with higher *δ*^18^O (*α*_0_ = 21.039). These results would suggest the relative higher influence of site on the depth of the preferred source water for cork rings formation, independently on the effect of the cork harvesting.

## Discussion

### Influence of water availability conditions on the *δ*^13^C of cork rings

Cork oak has the adaptive capacity to cope with variations in soil water availability during the growing season and can adjust its annual growth rhythm to prevailing environmental constrains, under the highly seasonal Mediterranean climate conditions (Cherubini et al., 2003; De Micco et al., 2016). Trees at Grândola (BS) typically present a bimodal growth pattern, as growth is subjected to a double constraint, by low temperatures in winter, and by drought in summer as in temperate climates (Cherubini et al., 2003). Generally, the onset of cork (and tree) growth occurs in spring, after a relative cold and wet winter, when soil water reserves have been replenished. In the summer, with limited water availability, trees experience a drought stress period and stop their stem (and cork) radial growth in July–August (summer rest period), resuming growth in autumn. However, trees at Benavente (CL) are able to reach the groundwater (Mendes et al., 2016), and to maintain their growth uninterruptedly from March to November; in fact, it is during the summer (June–July) that cork oaks’ radial growth (that it is mostly cork growth) reaches a maximum peak at this site (Costa, Pereira & Oliveira, 2003). So, different physiological growth patterns would be expected between the two sites.

Under low water availability (at BS), the higher cork rings *δ*^13^C, *i.e.,* cork-rings enriched in ^13^C (on average, −26.8‰ at BS against −27.3‰ at CL) (Fig. 3) would be mostly explained by a strong regulation (reduction) of stomatal conductance, possibly affecting photosynthesis, confirming our first hypothesis on that the cork rings *δ*^13^C variation may reflect the physiological and structural adjustments (*e.g.*, stomatal conductance) to cope with drought. The former values are slightly lower but in line with the range of values between −26.3‰ and −26.1‰ found in tree rings of the evergreen oak *Q. ilex* (Zalloni et al., 2018), where wood formation is determined by a double climate stress (summer drought and winter cold) and usually results from two phases of cambial activity separated by the summer rest period. Since cork mass is 40–50% suberin, a lipid-based polymer (Graça & Pereira, 2000; Graça, 2015), our results on ^13^C depleted cork-rings in comparison with tree-rings are in accordance with previous studies reporting that the metabolic pathway involved in lipid synthesis is much more ^13^C depleted than the one involved in (wood) cellulose synthesis. This occurs due to the ^13^C depletion of primary assimilates in the leaves to produce lipids, in contrast with ^13^C enrichment to produce cellulose (De Niro & Epstein, 1977; Melzer & Schmidt, 1987; Gessler & Ferrio, 2022). Moreover, our results on the correlations of cork rings *δ*^13^C with climate variables confirmed the critical role of temperature. This agrees with the fact that trees are forced to concentrate their photosynthetic activity in the warmer months of spring and autumn at both sites but mainly at the drier one (BS) (Fig. 4 and Table 2).

Noticeably, there is an apparent paradox in our results as trees at both sites: CL and BS, showed a general decreasing trend of *δ*^13^C in cork rings (*i.e.*,^13^C depleted cork rings) with increasing temperatures, and significant negative correlations between *δ*^13^C and mean temperature of the previous-year spring (Figs. 4A–4B). However, this depletion of ^13^C in cork rings can be explained by the possibility of the occurrence of post-photosynthetic processes associated with the remobilization and use of previous year(s) synthetized and stored carbon for cork formation, due to the shortage, or even the ^13^C discrimination of recent photo-assimilates in current (early) spring which are effectively ^13^C enriched (with higher *δ*^13^C) due to reduction of stomatal conductance. In cork oaks, a transfer of carbon reserves from the non-conducting to the conducting phloem tissue, close to the zone where phellogen is active, was reported by Knapic et al. (2007). These authors speculated that a displacement of these reserves in the form of extractives from heartwood to the outer sapwood for cork formation occurs at expenses of wood growth. It is possible that isotopic fractioning occurs during transport of metabolites As according to Cerasoli et al. (2004), the mobilization and utilization of stored carbon in cork oaks implies the hydrolysis of starch and the synthesis of sucrose, both enriched in ^13^C (Eglin et al., 2010) with a reduction of its concentration in stem wood. One can suggest that the preferential carbon reserves used for cork growth would be depleted in ^13^C, perhaps formed in less water stressed periods, and/or that the fractioning at the biochemical pathways of suberin synthesis would lead to compounds with very low *δ*^13^C (Damesin & Lelarge, 2003).

Trees at Benavente (CL) are with unlimited water availability and thus less water stressed. Cork growth should rely mainly on current photosynthates, relatively more depleted in ^13^C, in the onset of cork formation, in spring. Moreover, in autumn and warmer winters, trees use and probably store current photosynthates for the next season, which are less enriched in ^13^C (lower *δ*^13^C) given the lower tree water stress. In BS, relatively higher mean values of ^13^C in cork rings reflect limited water availability and trees water stress. Spring cork growth flush should rely not only on ^13^C enriched current photosynthates but on enriched ^13^C carbon reserves, which were stored during the previous dormant (winter) season (Ghalem et al., 2018), similarly to spring xylem (early wood) formed in tree-rings of other Mediterranean evergreen oaks, *e.g.*, *Q. ilex* (Ferrio et al., 2003; Gea-izquierdo, Cherubini & Cañellas, 2011; Shestakova et al., 2014; Zalloni et al., 2018), and even in deciduous *Q. petraea* (Barbaroux & Bréda, 2002).

Furthermore, suberin is the main structural compound of the cork cell walls and it has a very special biochemical composition, a polyester lipid build up from long chain (C16–C24) fatty acids, which have carboxylic acid and/or hydroxyl functionalities at both chain ends (Graça, 2015). When phellogen is active, the phellem cells undergo a relative quick normal sequential differential stage: expansion, massive deposition of suberin layers in the internal side of cell walls, and programmed cell death (Soler et al., 2007; Teixeira & Pereira, 2010; Inácio et al., 2018). Such construction and integration of suberin in cork could lead to a ^13^C depletion and a concomitant enrichment of ^13^C respired CO_2_, strongly and directly related with temperature, during cork formation as it is well known that respiratory processes discriminate against ^13^C.

Another possible explanation for the *δ*^13^C negative correlation with temperature is that, while the tight stomatal regulation is very responsive to water stress, as in a true drought-avoiding species, meaning that trees effectively display high stomatal resistance during drought, even reducing photosynthetic metabolism which may result in the growth impairment (Kurz-Besson et al., 2006), the rate of carboxylation and net photosynthesis would be much less sensitive or more complacent to water stress, which would decisively influence *δ*^13^C of carbohydrates, and lipids.

Overall, the particularly unexpected result on ^13^C isotopic signature in cork might reflect the dynamics of both processes, primary CO_2_ fixation (stomatal regulation) and the downstream metabolic processes, carbon photosynthetic assimilation or post-assimilation of carbon reserves, including the metabolites used for export and correspondent fractionation processes, at different times and under different environmental conditions. These are some alternative hypotheses which can potentially explain the varying rates of incorporation of relatively heavy carbon (^13^C) in suberin during cork formation, and which merits future research.

The examination through mixed modelling of cork rings *δ*^13^C variations between trees (random intercept model, M2) might help to understand the range of varying rates of ^13^C in cork. According to our results, cork rings at the drier site (BS), in the first year after cork harvesting (*y*_cork_ = 1) were clearly different and had less negative *δ*^13^C (^13^C enriched) than other cork-rings (*y*_cork_ = 6, at BS; or *y*_cork_ = 1 or 6, at CL) much more ^13^C depleted, despite sharing common variation in response to temperature (Table 2, Fig. 3). Recently harvest trees in BS are under severe water stress due to water loss from the cork harvested stem (and branches) which much exceeds the water lost by leaf transpiration during the summer season (Correia et al., 1992; Oliveira & Costa, 2012). This suggests that under these conditions, trees would close stomata, with the consequent reduction of CO_2_ concentration in the intercellular space, limiting CO_2_ availability for the carboxylation, which is then forced to less discrimination and fix a relative greater proportion of the heavier isotope (^13^C) (Farquhar et al., 1989). Also, water stress trigger suberin biosynthesis and suberization (Aguado et al., 2012; Boher et al., 2018), similarly to the promotion of xylogenesis in tree rings (Castagneri et al., 2018), and trees need to urgently regenerate their cork layers to survive. Under these conditions, only in the first year after cork harvest (*y*_cork_ = 1) and only at the drier site (BS), trees likely used mixing carbon pools in their metabolic pathways of suberin synthesis: from current assimilates (already relatively enriched in ^13^C) and from carbon reserves, which will be relatively more enriched in ^13^C, producing ^13^C enriched cork rings.

Despite the absence of significant correlations between cork ring parameters width (IC_RW_) and maximum density (IC_MD_) and *δ*^13^C isotopic composition, the way that trees generate cork cells or increase their walls density in response to climate conditions could be different, depending on the way they respond to water stress conditions and control stomata and/or photosynthetic activity. The IC_RW_ and IC_MD_ correlations with mean temperature (Table 1) were considerably weaker than the correlations found for *δ*^13^C (Fig. 4). This suggests that cork is formed, or its density is increased as a result of an overall tree performance on their biomass production, while ^13^C signatures reflect tree adaptive mechanisms of response to water stress and drought.

### Influence of water availability conditions on the *δ*^18^O of cork rings

Under a Mediterranean type climate, with water as the main limiting factor for plant growth, distinct cork *δ*^18^O should primarily reflect the variability in the water source used during growing season between trees. Furthermore, cork *δ*^18^O should also be responsive to the stomatal conductance, but not sensitive to photosynthetic activity, as the *δ*^13^C (Farquhar & Lloyd, 1993; Roden & Farquhar, 2012).

Cork rings *δ*^18^O revealed some differences between the two study areas. At drier site (BS) cork was more depleted in ^18^O than at the wetter site (CL), with higher *δ*^18^O (Fig. 5). Here, these *δ*^18^O higher values indicate mostly the use of a shallower, more isotopically enriched water source, but also probably some (leaf) water ^18^O enrichment, given the unlimited water availability suggesting a higher stomatal conductance (Fig. 5). Moreover, the spring March-May precipitation only affected the *δ*^18^O of cork rings at wetter site (CL) (Fig. 6C) suggesting some dependence on growing season precipitation for cork formation in the long and uninterrupted cork growth period (Costa, Pereira & Oliveira, 2003). Thus, relatively more enriched ^18^O cork-rings are expected.

In contrast, at the drier site (BS), cork-rings are more ^18^O depleted (Fig. 5). Moreover, no significant correlations were found with precipitation. These results might suggest that cork oak obtain their water from deeper soil horizons (Sarris, Christodoulakis & Körner, 2007), trees are less dependent on growing season precipitation (or even precipitation in winter previous to growth year) for cork formation and have a strong reliance on storage carbon reserves (Ferrio et al., 2015; Szymczak et al., 2019). Other possible explanation for the non-significant correlations with precipitation is that water reserves in deeper groundwater can represent a long-term mean isotopic value of winter precipitation and hence do not only reflect the amount of winter precipitation prior to the growth season (Szymczak et al., 2019). Furthermore, in contrast to CL, trees at BS have a stronger stomatal conductance regulation that probably would constrain the (leaf) water ^18^O enrichment.

Cork oak is a deep-rooted species, with a very effective water uptake from the seasonally recharged groundwater, and with a dual root system which allow to use of soil water, even in the drier summer season (Vaz et al., 2010; David et al., 2007). Moreover, the preferential water source of cork oak is deeper groundwater sources (Altieri et al., 2015). However, at BS, a direct comparison between *δ*^18^O of cork rings and seasonal fluctuations of precipitation can be hampered in several ways. First, seasonal cork (suberin) synthesis is decoupled from seasonal precipitation and distinct information can be encoded in the ^18^O of the cork rings; second, tree uses mixing pools of carbon which are formed with distinct water pools, for suberin synthesis in distinct growth seasons; third, depending on rooting depth, trees have to access to different water pools with varying isotopic signatures and it can be assumed that groundwater replenishment in winter previous to the growth season cannot be sufficient and trees depend also on precipitation during the growth season as other main water source.

Altogether, mixed modelling results points to the existence of a relationship between cork *δ*^18^O signatures and trees preferential water sources, confirming our second hypothesis, without the complexity of the effect of climate drivers and of the cork harvesting on the variability of *δ*^18^O in cork rings (Fig. 5; Table 2). However, further investigations are needed in order to clarify the interpretation of our results on the *δ*^18^O signature in cork rings.

## Conclusions

Our findings on the variability of stable isotopes *δ*^13^C and *δ*^18^O in cork rings from(cork) harvested trees growing under contrasting water availability conditions in Mediterranean environments revealed that *δ*^13^C might primarily reflect the regulation of trees’ stomatal conductance and photosynthetic activity under severe water stress, whereas *δ*^18^O primarily indicates the water sources used by the tree for cork growth. Cork formation is predominantly temperature-controlled, and tree uses carbon from mixing pools such as, previous year(s) synthetized and stored carbon or recent photo-assimilated carbon, in the metabolic pathways of suberin synthesis which seemed to highly discriminate ^13^C. Further studies would be necessary to elucidate the origin of carbon reserves, post carbonylation fractionating or carbon mobilization and the influence of environmental conditions on these processes.

The findings of this exploratory study show the potential of addressing the physiological implications of water stress for cork oak (or cork) growth through the isotope signature in cork rings, thus facilitating the evaluation of its relevance in climate change scenarios for site-specific tree adaptation.

##  Supplemental Information

10.7717/peerj.14270/supp-1 Table S1 Location of trees used for cork sampling Coordinates (LAT and LONG) of the trees sampled for cork in the study areas in Benavente (CL) and in Grândola (BS)

10.7717/peerj.14270/supp-2 Table S2 Pearson’s correlations coefficient (*r*) between climate parameters (precipitation and temperature and cork-ring’s *δ*^13^C)

10.7717/peerj.14270/supp-3 Table S3 Pearson’s correlations coefficient (*r*) between climate parameters (mean temperature and precipitation and cork-ring’s *δ*^18^O)

10.7717/peerj.14270/supp-4 Data S1 Cork-ring’s isotopic composition raw data Each record indicates the average isotopic composition in cork-rings of stable carbon isotope ratio (*d*^13^C)—d13Cc and stable oxygen isotope ratio (*d*^18^O)—d18Oc per Site × Tree × Year. Also, raw data of oxygen isotope ratio (*d*^18^O) of suberin—d18Oc is presented. This later dataset were not used in this study.

## Additional Information and Declarations

### Data Availability

The following information was supplied regarding data availability: The raw data are available in the Supplemental Files.

## References

[ref-1] Aguado PL, Curt MD, Pereira H, Fernandez J (2012). Allocation of C-14 assimilated in late spring to tissue and biochemical stem components of cork oak (*Quercus suber* L.) over the seasons. Tree Physiology.

[ref-2] Altieri S, Mereu S, Cherubini P, Castaldi S, Sirignano C, Lubritto C, Battipaglia G (2015). Tree-ring carbon and oxygen isotopes indicate different water use strategies in three Mediterranean shrubs at Capo Caccia (Sardinia, Italy). Trees—Structure and Function.

[ref-3] Araguás-Araguás L, Froehlich K, Rozanski K (2000). Deuterium and oxygen-18 isotope composition of precipitation and atmospheric moisture. Hydrological Processes.

[ref-4] Barbaroux C, Bréda N (2002). Contrasting distribution and seasonal dynamics of carbohydrate reserves in stem wood of adult ring-porous sessile oak and diffuse-porous beech trees. Tree Physiology.

[ref-5] Barbour MM (2007). Stable oxygen isotope composition of plant tissue: a review. Functional Plant Biology.

[ref-6] Barbour MM, Walcroft AS, Farquhar GD (2002). Seasonal variation in delta C-13 and delta O-18 of cellulose from growth rings of *Pinus radiata*. Plant, Cell & Environment.

[ref-7] Boher P, Soler M, Sánchez A, Hoede C, Noirot C, Paiva JAP, Serra O, Figueras M (2018). A comparative transcriptomic approach to understanding the formation of cork. Plant Molecular Biology.

[ref-8] Caritat A, Gutiérrez E, Molinas M (2000). Influence of weather on cork-ring width. Tree Physiology.

[ref-9] Castagneri D, Battipaglia G, Von Arx G, Pacheco A, Carrer M (2018). Tree-ring anatomy and carbon isotope ratio show both direct and legacy effects of climate on bimodal xylem formation in *Pinus pinea*. Tree Physiology.

[ref-10] Cerasoli S, Maillard P, Scartazza A, Brugnoli E, Chaves MM, Pereira JS (2004). Carbon and nitrogen winter storage and remobilisation during seasonal flush growth in two-year-old cork oak (*Quercus suber* L.) saplings. Annals of Forest Science.

[ref-11] Cherubini P, Battipaglia G, Innes JL (2021). Tree vitality and forest health: can tree-ring stable isotopes be used as indicators?. Current Forestry Reports.

[ref-12] Cherubini P, Gartner BL, Tognetti R, Bräker OU, Schoch W, Innes JL (2003). Identification, measurement and interpretation of tree rings in woody species from Mediterranean climates. Biological Reviews.

[ref-13] Correia OA, Oliveira G, Martins-Loução MA, Catarino FM (1992). Effects of bark stripping on the water relations of *Quercus suber* L. Scientia Gerundensis.

[ref-14] Costa A, Barbosa I, Pestana M, Célia M (2020). Modelling bark thickness variation in stems of cork oak in south-western Portugal. European Journal of Forest Research.

[ref-15] Costa A, Barbosa I, Roussado C, Graça J, Spiecker H (2016). Climate response of cork growth in Mediterranean oak (*Quercus suber* L.) woodlands of southwestern Portugal. Dendrochronologia.

[ref-16] Costa A, Cherubini P (2021). Is cork growth a reliable proxy for stem diameter growth in cork oak (*Quercus suber* L.)? Implications for forest management under climate change in Mediterranean regions. Applied Sciences.

[ref-17] Costa A, Graça J, Barbosa I, Spiecker H (2022). Effect of climate on cork-ring width and density of *Quercus suber* L. in southern Portugal. Trees.

[ref-18] Costa A, Madeira M, Oliveira AC (2008). The relationship between cork oak growth patterns and soil, slope and drainage in a cork oak woodland in Southern Portugal. Forest Ecology and Management.

[ref-19] Costa A, Nunes LC, Spiecker H, Graça J (2015). Insights into the cork oak (*Quercus suber* L.) responsiveness to bark harvesting. Economic Botany.

[ref-20] Costa A, Pereira H, Oliveira A (2002). Influence of climate on the seasonality of radial growth of cork oak during a cork production cycle. Annals of Forest Science.

[ref-21] Costa A, Pereira H, Oliveira A (2003). Variability of radial growth in cork oak adult trees under cork production. Forest Ecology and Management.

[ref-22] Damesin C, Lelarge C (2003). Carbon isotope composition of current year shoots from Fagus sylvatica in relation to growth, respiration and use of reserves. Plant, Cell & Environment.

[ref-23] David TS, Henriques MO, Kurz-Besson C, Nunes J, Valente F, Vaz M, Pereira JS, Siegwolf R, Chaves MM, Gazarini LC, David JS (2007). Water-use strategies in two co-occurring Mediterranean evergreen oaks: surviving the summer drought. Tree Physiology.

[ref-24] De Micco V, Balzano A, Čufar K, Aronne G, Gričar J, Merela M, Battipaglia G (2016). Timing of false ring formation in *Pinus halepensis* and *Arbutus unedo* in southern Italy: outlook from an analysis of xylogenesis and tree-ring chronologies. Frontiers in Plant Science.

[ref-25] De Niro MJ, Epstein S (1977). Mechanisms of carbon isotope fractionation associated with lipid-synthesis. Science.

[ref-26] Eglin T, Francois C, Michelot A, Delpierre N, Damesin C (2010). Linking intra-seasonal variations in climate and tree-ring *δ*^13^C: a functional modelling approach. Ecological modelling.

[ref-27] EUFORGEN (2019). Distribution map of cork oak (*Quercus suber* L.). European forest genetic resources programme—EUFORGEN, Rome, Italy, web site. [online].

[ref-28] FAO (2014). State of the world’s forests. Enhancing the socioeconomic benefits from forests. http://www.fao.org/3/i3710e/i3710e.pdf.

[ref-29] Farquhar GD, Hubick KT, Condon AG, Richards RA, Rundel PW, Ehleringer JR, Nagy KA (1989). Carbon isotope discrimination and water-use efficiency. Stable isotopes in ecological research.

[ref-30] Farquhar GD, Lloyd J, Ehleringer JR, Hall AE, Farquhar GD (1993). Carbon and oxygen isotope effects in the exchange of carbon dioxide between terrestrial plants and the atmosphere. Stable isotopes and plant carbon–water relations.

[ref-31] Ferrio JP, Díez-Herrero A, Tarrés D, Ballesteros-Cánovas JA, Aguilera M, Bodoque JM (2015). Using stable isotopes of oxygen from tree-rings to study the origin of past flood events: first results from the Iberian Peninsula. Quaternaire.

[ref-32] Ferrio JP, Florit A, Vega A, Serrano L, Voltas J (2003). d13C and tree-ring width reflect different drought responses in *Quercus ilex* and *Pinus halepensis*. Oecologia.

[ref-33] Garzón MB, Dios RSánchezde, Ollero HS (2008). Effects of climate change on the distribution of Iberian tree species. Applied Vegetation Science.

[ref-34] Gea-izquierdo G, Cherubini P, Cañellas I (2011). Tree-rings reflect the impact of climate change on *Quercus ilex* L. along a temperature gradient in Spain over the last 100 years. Forest Ecology and Management.

[ref-35] Gessler A, Ferrio JP, Siegwolf RTW, Brooks JR, Roden J, Saurer M (2022). Postphotosynthetic fractionation in leaves, phloem and stem. Stable isotopes in tree rings. Inferring physiological, climatic and environmental responses.

[ref-36] Gessler A, Ferrio JP, Hommel R, Treydte K, Werner RA, Monson RK (2014). Stable isotopes in tree rings: towards a mechanistic understanding of isotope fractionation and mixing processes from the leaves to the wood. Tree Physiology.

[ref-37] Ghalem A, Barbosa I, Bouhroua RT, Costa A (2018). Climate signal in cork-rings chronologies: case-studies on southwestern Portugal and north-western Algeria. Tree-Ring Research.

[ref-38] Graça J (2015). Suberin: the biopolyester at the frontier of plants. Frontiers in Chemistry.

[ref-39] Graça J, Pereira H (2000). Methanolysis of bark suberins: analysis of glycerol and acid monomers. Phytochemical Analysis.

[ref-40] Hafner P, Gričar J, Skudnik M, Levanič T (2015). Variations in environmental signals in tree-ring indices in trees with different growth potential. PLOS ONE.

[ref-41] Inácio V, Martins MT, Graça J, Morais-Cecílio L (2018). Cork oak young and traumatic periderms show PCD typical chromatin patterns but different chromatin-modifying genes expression. Frontiers in Plant Science.

[ref-42] Shukla PRJ, Skea R, Van Diemen K, Calvin Ø, Christophersen F, Creutzig J, IPCC (2017). Meeting report of the intergovernmental panel on climate change expert meeting on mitigation, sustainability and climate stabilization scenarios.

[ref-43] Knapic S, Louzada JL, Leal S, Pereira H (2007). Radial variation of wood density components and ring width in cork oak trees. Annals of Forest Science.

[ref-44] Kurz-Besson C, Otieno D, Vale RL, Siegwolf R, Schmidt M, Herd A, Nogueira C, David TS, David JS, Tenhunen J, Pereira JS, Chaves M (2006). Hydraulic lift in cork oak trees in a savannah-type mediterranean ecosystem and its contribution to the local water balance. Plant Soil.

[ref-45] Lebourgeois F (2000). Climatic signals in earlywood, latewood and total ring width of Corsican pine from western France. Annals of Forest Science.

[ref-46] Leite C, Oliveira V, Miranda I, Pereira H (2020). Cork oak and climate change: disentangling drought effects on cork chemical composition. Scientific Reports.

[ref-47] Loader NJ, Robertson I, McCarroll D (2003). Comparison of stable carbon isotope ratios in the whole wood, cellulose and lignin of oak tree-rings. Palaeogeography, Palaeoclimatology, Palaeoecology.

[ref-48] Loader NJ, Santillo PM, Woodman-Ralph JP, Rolfe JE, Hall MA, Gagen M, Robertson I, Wilson R, Froyd CA, McCarroll D (2008). Multiple stable isotopes from oak trees in southwestern Scotland and the potential for stable isotope dendroclimatology in maritime climatic regions. Chemical Geology.

[ref-49] McCarroll D, Loader NJ (2004). Stable isotopes in tree rings. Quaternary Science Reviews.

[ref-50] Melzer E, Schmidt HL (1987). Carbon isotope effects on the pyruvate dehydrogenase reaction and their importance for relative carbon-13 depletion in lipids. Journal of Biological Chemistry.

[ref-51] Mendes MP, Cherubini P, Plieninger T, Ribeiro L, Costa A (2019). Climate effects on stem radial growth of *Quercus suber* L.: does tree size matter?. Forestry.

[ref-52] Mendes MP, Ribeiro L (2010). Nitrate probability mapping in the northern aquifer alluvial system of the river Tagus (Portugal) using Disjunctive Kriging. Science of the Total Environment.

[ref-53] Mendes MP, Ribeiro L, David TS, Costa A (2016). How dependent are cork oak (*Quercus suber* L.) woodlands on groundwater? A case study in southwestern Portugal. Forest Ecology and Management.

[ref-54] O’Leary MH (1988). Carbon isotopes in photosynthesis. Fractionation techniques may reveal new aspects of carbon dynamics in plants. Bioscience.

[ref-55] Oliveira G, Correia O, Martins-Loução MA, Catarino FM (1994). Phenological and growth patterns of the Mediterranean oak Quercus suber L. Trees—Structure and Function.

[ref-56] Oliveira G, Costa A (2012). How resilient is *Quercus suber* L. to cork harvesting? A review and identification of knowledge gaps. Forest Ecology and Management.

[ref-57] Oliveira V, Lauw A, Pereira H (2016). Sensitivity of cork growth to drought events: insights from a 24-year chronology. Climatic Change.

[ref-58] Pereira H (2007). Cork: biology, production and uses.

[ref-59] R Development Core Team (2019). https://www.r-project.org.

[ref-60] Roden JS, Farquhar GD (2012). A controlled test of the dual-isotope approach for the interpretation of stable carbon and oxygen isotope ratio variation in tree rings. Tree Physiology.

[ref-61] Sarris D, Christodoulakis D, Körner C (2007). Recent decline in precipitation and tree growth in the eastern Mediterranean. Global Change Biology.

[ref-62] Saurer M, Cherubini P, Reynolds-Henne CE, Treydte KS, Anderson WT, Siegwolf RTW (2008). An investigation of the common signal in tree ring stable isotope chronologies at temperate sites. Journal of Geophysical Research.

[ref-63] Scheidegger Y, Saurer M, Bahn M, Siegwolf R (2000). Linking stable oxygen and carbon isotopes with stomatal conductance and photosynthetic capacity: a conceptual model. Oecologia.

[ref-64] Shestakova TA, Aguilera M, Ferrio JP, Gutiérrez E, Voltas J (2014). Unravelling spatiotemporal tree-ring signals in Mediterranean oaks: a variance–covariance modelling approach of carbon and oxygen isotope ratios. Tree Physiology.

[ref-65] Soler M, Serra O, Molinas M, Huguet G, Fluch S, Figueras M (2007). A genomic approach to suberin biosynthesis and cork differentiation. Plant Physiology.

[ref-66] Song X, Clark KS, Helliker BR (2014). Interpreting species-specific variation in tree-ring oxygen isotope ratios among three temperate forest trees. Plant, Cell & Environment.

[ref-67] Sternberg L, Ellsworth PFV (2011). Divergent biochemical fractionation, not convergent temperature, explains cellulose oxygen isotope enrichment across latitudes. PLOS ONE.

[ref-68] Szymczak S, Bräuning A, Häusser M, Garel E, Huneau F, Santoni S (2019). The relationship between climate and the intra-annual oxygen isotope patterns from pine trees: a case study along an elevation gradient on Corsica, France. Annals of Forest Science.

[ref-69] Teixeira RT, Pereira H (2010). Suberized cell walls of cork from cork oak differ from other species. Microscopy and Microanalysis.

[ref-70] Vaz M, Pereira JS, Gazarini LC, David TS, David JS, Rodrigues A, Maroco J, Chaves MM (2010). Drought-induced photosynthetic inhibition and autumn recovery in two Mediterranean oak species (*Quercus ilex* and *Quercus suber*). Tree Physiology.

[ref-71] Zalloni E, Battipaglia G, Cherubini P, Saurer M, De Micco V (2018). Contrasting physiological responses to Mediterranean climate variability are revealed by intra-annual density fluctuations in tree rings of *Quercus ilex* L. and *Pinus pinea* L. Tree Physiology.

