# Supplementary material for: Beyond width and density: stable carbon and oxygen isotopes in cork-rings provide insights of physiological responses to water stress in Quercus suber L
Source: PeerJ. 2022 Nov 14;10:e14270. doi: 10.7717/peerj.14270 (PMC9671033; doi:10.7717/peerj.14270)
Supplement: Table S1 — Coordinates (LAT and LONG) of the trees sampled for cork in the study areas in Benavente (CL) and in Grândola (BS) [file peerj-10-14270-s001.zip › Table S1_Supplementary_Material_Trees Coordinates.docx]

Table S1: Coordinates of the trees sampled for cork in the study areas in Benavente (CL) and in Grândola (BS)

| Tree Code | LAT | LON | Period of measurements | Climate period |
| --- | --- | --- | --- | --- |
| CL2 | 38°50'8.65"N | 8°49'7.31"W | 1968 - 2013 | 1967-2013 |
| CL4 | 38°50'6.41"N | 8°49'5.57"W | 1968 - 2013 | 1967-2013 |
| CL44 | 38°50'3.49"N | 8°49'5.25"W | 1964-2012 | 1963 - 2012 |
| CL49 | 38°50'6.33"N | 8°49'8.50"W | 1964-2012 | 1963 - 2012 |
| BS1 | 38°11'48.26"N | 8°36'13.69"W | 1962 - 2012 | 1961-2012 |
| BS2 | 38°11'47.48"N | 8°36'16.69"W | 1962 - 2012 | 1961-2012 |
| BS5 | 38°11'46.54"N | 8°36'13.98"W | 1962 - 2012 | 1961-2012 |
| BS8 | 38°11'43.86"N | 8°36'11.53"W | 1962 - 2012 | 1961-2012 |
