# Supplementary material for: Beyond width and density: stable carbon and oxygen isotopes in cork-rings provide insights of physiological responses to water stress in Quercus suber L
Source: PeerJ. 2022 Nov 14;10:e14270. doi: 10.7717/peerj.14270 (PMC9671033; doi:10.7717/peerj.14270)
Supplement: Table S2 [file peerj-10-14270-s002.zip › Table S2_Supplementary_Material_Correlations_Climate_CarbonIsotope.docx]

Table S2: A) Study areas Benavente (CL) and Grândola (BS). Pearson’s correlations coefficient ( r ) between Mean Temperature and cork-ring’s δ^13^C. p-value<0.01 (**); p-value < 0.05 (*); p-value> 0.05 (non-significant at alpha level of 0.05, n.s.)

| Period (months) | Benavente - CL (n= 34) | | Grândola - BS (n=44) | |
| --- | --- | --- | --- | --- |
|  | Pearson's Correlation coefficient ( r) | p-value | Pearson's Correlation coefficient ( r) | p-value |
| Feb | -0.199 | n.s. | **-0.393** | ****** |
| Mar | -0.243 | n.s. | **-0.339** | ***** |
| Apr | **-0.451** | ****** | -0.284 | n.s. |
| May | **-0.370** | ***** | -0.197 | n.s. |
| Jun | 0.043 | n.s. | -0.039 | n.s. |
| Jul | -0.011 | n.s. | -0.272 | n.s. |
| Aug | -0.233 | n.s. | -0.289 | n.s. |
| Sep | -0.223 | n.s. | -0.140 | n.s. |
| Oct | -0.067 | n.s. | 0.073 | n.s. |
| Nov | **-0.431** | ***** | -0.263 | n.s. |
| Dec | -0.129 | n.s. | **-0.302** | ***** |
| JAN | -0.166 | n.s. | -0.222 | n.s. |
| FEB | -0.179 | n.s. | -0.216 | n.s. |
| MAR | -0.302 | n.s. | **-0.371** | ***** |
| APR | -0.064 | n.s. | -0.180 | n.s. |
| MAY | -0.182 | n.s. | -0.138 | n.s. |
| JUN | -0.125 | n.s. | **-0.315** | ***** |
| JUL | -0.256 | n.s. | -0.192 | n.s. |
| AUG | -0.178 | n.s. | **-0.312** | ***** |
| SEP | 0.058 | n.s. | -0.247 | n.s. |
| OCT | -0.056 | n.s. | -0.038 | n.s. |
| NOV | -0.247 | n.s. | -0.180 | n.s. |
| Feb | -0.199 | n.s. | **-0.393** | ****** |
| Feb-Mar | -0.243 | n.s. | **-0.401** | ****** |
| Feb-Apr | -0.328 | n.s. | **-0.385** | ****** |
| Feb-May | **-0.364** | ***** | **-0.396** | ****** |
| Feb-Jun | **-0.355** | ***** | **-0.397** | ****** |
| Feb-Jul | -0.303 | n.s. | **-0.415** | ****** |
| Feb-Aug | -0.305 | n.s. | **-0.413** | ****** |
| Feb-Sep | -0.303 | n.s. | **-0.423** | ****** |
| Feb-Oct | -0.290 | n.s. | **-0.384** | ***** |
| Feb-Nov | -0.276 | n.s. | **-0.374** | ***** |
| Feb-Dec | -0.265 | n.s. | **-0.379** | ***** |
| Feb-JAN | -0.267 | n.s. | **-0.372** | ***** |
| Mar | -.243 | n.s. | **-0.339** | ***** |
| Mar-Apr | **-0.358** | ***** | **-0.336** | ***** |
| Mar-May | **-0.395** | ***** | **-0.355** | ***** |
| Mar-Jun | **-0.379** | ***** | **-0.349** | ***** |
| Mar-Jul | -0.312 | n.s. | **-0.380** | ***** |
| Mar-Aug | -0.311 | n.s. | **-0.380** | ***** |
| Mar-Sep | -0.307 | n.s. | **-0.392** | ****** |
| Mar-Oct | -0.291 | n.s. | **-0.352** | ***** |
| Mar-Nov | -0.265 | n.s. | **-0.346** | ***** |
| Mar-Dec | -0.256 | n.s. | **-0.358** | ***** |
| Mar-JAN | -0.259 | n.s. | **-0.354** | ***** |
| Mar-FEB | -0.259 | n.s. | **-0.354** | ***** |
| Apr | **-0.451** | ****** | -0.284 | n.s. |
| Apr-May | **-0.462** | ****** | **-0.303** | ***** |
| Apr-Jun | **-0.393** | ***** | -0.288 | n.s. |
| Apr-Jul | -0.297 | n.s. | **-0.338** | ***** |
| Apr-Aug | -0.294 | n.s. | **-0.340** | ***** |
| Apr-Sep | -0.295 | n.s. | **-0.356** | ***** |
| Apr-Oct | -0.277 | n.s. | **-0.318** | ***** |
| Apr-Nov | -0.244 | n.s. | **-0.317** | ***** |
| Apr-Dec | -0.239 | n.s. | **-0.338** | ***** |
| Apr-JAN | -0.247 | n.s. | **-0.337** | ***** |
| Apr-FEB | -0.250 | n.s. | **-0.341** | ***** |
| Apr-MAR | -0.262 | n.s. | **-0.356** | ***** |
| May | **-0.370** | ***** | -0.197 | n.s. |
| May-Jun | -0.182 | n.s. | -0.163 | n.s. |
| May-Jul | -0.128 | n.s. | -0.239 | n.s. |
| May-Aug | -0.166 | n.s. | -0.262 | n.s. |
| May-Sep | -0.198 | n.s. | -0.279 | n.s. |
| May-Oct | -0.192 | n.s. | -0.257 | n.s. |
| May-Nov | -0.156 | n.s. | -0.280 | n.s. |
| May-Dec | -0.171 | n.s. | **-0.313** | * |
| May-JAN | -0.191 | n.s. | **-0.318** | ***** |
| May-FEB | -0.204 | n.s. | **-0.327** | ***** |
| May-MAR | -0.223 | n.s. | **-0.347** | ***** |
| May-APR | -0.213 | n.s. | **-0.346** | * |
| Jun | 0.043 | n.s. | -0.039 | n.s. |
| Jun-Jul | 0.022 | n.s. | -0.176 | n.s. |
| Jun-Aug | -0.071 | n.s. | -0.236 | n.s. |
| Jun-Sep | -0.128 | n.s. | -0.257 | n.s. |
| Jun-Oct | -0.129 | n.s. | -0.228 | n.s. |
| Jun-Nov | -0.088 | n.s. | -0.266 | n.s. |
| Jun-Dec | -0.121 | n.s. | **-0.299** | ***** |
| Jun-JAN | -0.151 | n.s. | **-0.306** | ***** |
| Jun-FEB | -0.169 | n.s. | **-0.312** | ***** |
| Jun-MAR | -0.193 | n.s. | **-0.333** | ***** |
| Jun-APR | -0.186 | n.s. | **-0.335** | ***** |
| Jun-MAY | -0.190 | n.s. | **-0.332** | ***** |
| Jul | -0.011 | n.s. | -0.272 | n.s. |
| Jul-Aug | -0.129 | n.s. | **-0.309** | ***** |
| Jul-Sep | -0.177 | n.s. | **-0.308** | ***** |
| Jul-Oct | -0.166 | n.s. | -0.243 | n.s. |
| Jul-Nov | -0.102 | n.s. | -0.273 | n.s. |
| Jul-Dec | -0.140 | n.s. | **-0.304** | ***** |
| Jul-JAN | -0.169 | n.s. | **-0.308** | ***** |
| Jul-FEB | -0.183 | n.s. | **-0.309** | ***** |
| Jul-MAR | -0.206 | n.s. | **-0.332** | ***** |
| Jul-APR | -0.199 | n.s. | **-0.337** | ***** |
| Jul-MAY | -0.202 | n.s. | **-0.336** | ***** |
| Jul-JUN | -0.203 | n.s. | **-0.358** | ***** |
| Aug | -0.233 | n.s. | -0.289 | n.s. |
| Aug-Sep | -0.246 | n.s. | -0.274 | n.s. |
| Aug-Oct | -0.203 | n.s. | -0.186 | n.s. |
| Aug-Nov | -0.127 | n.s. | -0.248 | n.s. |
| Aug-Dec | -0.169 | n.s. | -0.293 | n.s. |
| Aug-JAN | -0.192 | n.s. | -0.295 | n.s. |
| Aug-FEB | -0.202 | n.s. | -0.297 | n.s. |
| Aug-MAR | -0.223 | n.s. | **-0.323** | ***** |
| Aug-APR | -0.215 | n.s. | **-0.330** | ***** |
| Aug-MAY | -0.217 | n.s. | **-0.330** | ***** |
| Aug-JUN | -0.217 | n.s. | **-0.355** | ***** |
| Aug-JUL | -0.228 | n.s. | **-0.350** | ***** |
| Sep | -0.223 | n.s. | -0.140 | n.s. |
| Sep-Oct | -0.157 | n.s. | -0.025 | n.s. |
| Sep-Nov | -0.086 | n.s. | -0.183 | n.s. |
| Sep-Dec | -0.155 | n.s. | -0.258 | n.s. |
| Sep-JAN | -0.183 | n.s. | -0.267 | n.s. |
| Sep-FEB | -0.194 | n.s. | -0.269 | n.s. |
| Sep-MAR | -0.217 | n.s. | **-0.302** | ***** |
| Sep-APR | -0.209 | n.s. | **-0.313** | ***** |
| Sep-MAY | -0.211 | n.s. | **-0.316** | ***** |
| Sep-JUN | -0.210 | n.s. | **-0.346** | ***** |
| Sep-JUL | -0.222 | n.s. | **-0.341** | ***** |
| Sep-AUG | -0.217 | n.s. | **-0.359** | ***** |
| Oct | -.067 | n.s. | .073 | n.s. |
| Oct-Nov | -.041 | n.s. | -.139 | n.s. |
| Oct-Dec | -.161 | n.s. | -.224 | n.s. |
| Oct-JAN | -.189 | n.s. | -.237 | n.s. |
| Oct-FEB | -.196 | n.s. | -.247 | n.s. |
| Oct-MAR | -.220 | n.s. | -.283 | n.s. |
| Oct-APR | -.209 | n.s. | -.295 | n.s. |
| Oct-MAY | -.210 | n.s. | **-0.298** | ***** |
| Oct-JUN | -.208 | n.s. | **-0.327** | ***** |
| Oct-JUL | -.221 | n.s. | **-0.324** | ***** |
| Oct-AUG | -.215 | n.s. | **-0.344** | ***** |
| Oct-SEP | -.207 | n.s. | **-0.349** | ***** |
| Nov | **-0.431** | ***** | -0.263 | n.s. |
| Nov-Dec | -0.280 | n.s. | **-0.315** | ***** |
| Nov-JAN | -0.251 | n.s. | **-0.303** | ***** |
| Nov-FEB | -0.235 | n.s. | -0.297 | n.s. |
| Nov-MAR | -0.250 | n.s. | **-0.330** | ***** |
| Nov-APR | -0.232 | n.s. | **-0.337** | ***** |
| Nov-MAY | -0.230 | n.s. | **-0.336** | ***** |
| Nov-JUN | -0.226 | n.s. | **-0.362** | ***** |
| Nov-JUL | -0.235 | n.s. | **-0.358** | ***** |
| Nov-AUG | -0.226 | n.s. | **-0.375** | ***** |
| Nov-SEP | -0.217 | n.s. | **-0.375** | ***** |
| Nov-OCT | -0.329 | n.s. | **-0.375** | ***** |
| Dec | -0.129 | n.s. | **-0.302** | ***** |
| Dec-JAN | -0.162 | n.s. | -0.291 | n.s. |
| Dec-FEB | -0.174 | n.s. | -0.195 | n.s. |
| Dec-MAR | -0.211 | n.s. | **-0.326** | ***** |
| Dec-APR | -0.199 | n.s. | **-0.335** | ***** |
| Dec-MAY | -0.211 | n.s. | **-0.331** | ***** |
| Dec-JUN | -0.218 | n.s. | **-0.365** | ***** |
| Dec-JUL | -0.238 | n.s. | **-0.359** | ***** |
| Dec-AUG | -0.234 | n.s. | **-0.377** | ***** |
| Dec-SEP | -0.228 | n.s. | **-0.375** | ***** |
| Dec-OCT | -0.344 | n.s. | **-0.375** | ***** |
| Dec-NOV | -0.344 | n.s. | **-0.367** | ***** |
| JAN-FEB | -0.178 | n.s. | -0.238 | n.s. |
| JAN-MAR | -0.228 | n.s. | **-0.311** | ***** |
| JAN-APR | -0.213 | n.s. | **-0.331** | ***** |
| JAN-MAY | -0.227 | n.s. | **-0.330** | ***** |
| JAN-JUN | -0.231 | n.s. | **-0.375** | ***** |
| JAN-JUL | -0.249 | n.s. | **-0.366** | ***** |
| JAN-AUG | -0.242 | n.s. | **-0.386** | ***** |
| JAN-SEP | -0.237 | n.s. | **-0.380** | ***** |
| JAN-OCT | **-0.354** | ***** | **-0.379** | ***** |
| JAN-NOV | **-0.352** | ***** | **-0.370** | ***** |
| FEB-MAR | -0.253 | n.s. | **-0.324** | ***** |
| FEB-APR | -0.220 | n.s. | **-0.347** | ***** |
| FEB-MAY | -0.234 | n.s. | **-0.328** | ***** |
| FEB-JUN | -0.233 | n.s. | **-0.38** | ***** |
| FEB-JUL | -0.255 | n.s. | **-0.366** | ***** |
| FEB-AUG | -0.244 | n.s. | **-0.379** | ***** |
| FEB-SEP | -0.238 | n.s. | **-0.372** | ***** |
| FEB-OCT | -0.340 | n.s. | **-0.373** | ***** |
| FEB-NOV | -0.340 | n.s. | **-0.366** | ***** |
| MAR-APR | -0.206 | n.s. | **-0.353** | ***** |
| MAR-MAY | -0.221 | n.s. | **-0.298** | ***** |
| MAR-JUN | -0.216 | n.s. | **-0.349** | ***** |
| MAR-JUL | -0.254 | n.s. | **-0.342** | ***** |
| MAR-AUG | -0.242 | n.s. | **-0.361** | ***** |
| MAR-SEP | -0.234 | n.s. | **-0.360** | ***** |
| MAR-OCT | -0.306 | n.s. | **-0.361** | ***** |
| MAR-NOV | -0.311 | n.s. | **-0.357** | ***** |
| APR-MAY | -0.132 | n.s. | -0.195 | n.s. |
| APR-JUN | -0.149 | n.s. | -0.297 | n.s. |
| APR-JUL | -0.220 | n.s. | **-0.305** | ***** |
| APR-AUG | -0.215 | n.s. | **-0.339** | ***** |
| APR-SEP | -0.207 | n.s. | **-0.339** | ***** |
| APR-OCT | -0.295 | n.s. | **-0.339** | ***** |
| APR-NOV | -0.304 | n.s. | **-0.338** | ***** |
| MAY-JUN | -0.178 | n.s. | -0.284 | n.s. |
| MAY-JUL | -0.250 | n.s. | -0.287 | n.s. |
| MAY-AUG | -0.237 | n.s. | **-0.321** | ***** |
| MAY-SEP | -0.229 | n.s. | **-0.320** | ***** |
| MAY-OCT | -0.299 | n.s. | **-0.322** | ***** |
| MAY-NOV | -0.309 | n.s. | **-0.323** | ***** |
| JUN-JUL | -0.238 | n.s. | -0.327 | * |
| JUN-AUG | -0.229 | n.s. | -0.360 | * |
| JUN-SEP | -0.216 | n.s. | -0.347 | * |
| JUN-OCT | -0.296 | n.s. | -0.347 | * |
| JUN-NOV | -0.306 | n.s. | -0.349 | * |
| JUL-AUG | -0.237 | n.s. | -0.290 | n.s. |
| JUL-SEP | -0.219 | n.s. | -0.291 | n.s. |
| JUL-OCT | -0.318 | n.s. | -0.293 | n.s. |
| JUL-NOV | -0.316 | n.s. | -0.295 | n.s. |
| AUG-SEP | -0.124 | n.s. | -0.298 | n.s. |
| AUG-OCT | -0.170 | n.s. | **-0.307** | ***** |
| AUG-NOV | -0.219 | n.s. | **-0.316** | ***** |
| SEP-OCT | 0.001 | n.s. | -0.221 | n.s. |
| SEP-NOV | -0.138 | n.s. | -0.251 | n.s. |
| OCT-NOV | -0.180 | n.s. | -0.142 | n.s. |

Table S2: B) Study areas: Benavente (CL) and Grândola (BS). Pearson’s correlations coefficient ( r ) between Precipitation and cork-ring’s δ^13^C. p-value < 0.05 (*); p-value> 0.05 (non-significant at alpha level of 0.05, n.s.)

| Period (months) | Benavente - CL (n= 34) | | Grândola - BS (n=44) | |
| --- | --- | --- | --- | --- |
|  | Pearson's Correlation coefficient ( r) | p-value | Pearson's Correlation coefficient ( r) | p-value |
| Feb | 0.191 | n.s. | 0.157 | n.s. |
| Mar | 0.130 | n.s. | 0.289 | n.s. |
| Apr | 0.023 | n.s. | -0.251 | n.s. |
| May | -0.142 | n.s. | -0.138 | n.s. |
| Jun | 0.040 | n.s. | -0.044 | n.s. |
| Jul | -0.248 | n.s. | -0.057 | n.s. |
| Aug | 0.010 | n.s. | -0.130 | n.s. |
| Sep | 0.185 | n.s. | -0.171 | n.s. |
| Oct | -0.075 | n.s. | -0.036 | n.s. |
| Nov | -0.096 | n.s. | -0.200 | n.s. |
| Dec | 0.200 | n.s. | 0.009 | n.s. |
| JAN | -0.118 | n.s. | 0.043 | n.s. |
| FEB | 0.165 | n.s. | 0.084 | n.s. |
| MAR | **0.398** | ***** | 0.023 | n.s. |
| APR | -0.099 | n.s. | -0.287 | n.s. |
| MAY | -0.089 | n.s. | -0.162 | n.s. |
| JUN | -0.0679 | n.s. | **0.324** | ***** |
| JUL | 0.202 | n.s. | 0.232 | n.s. |
| AUG | -0.263 | n.s. | -0.087 | n.s. |
| SEP | -0.102 | n.s. | -0.168 | n.s. |
| OCT | 0.096 | n.s. | 0.021 | n.s. |
| NOV | 0.103 | n.s. | 0.252 | n.s. |
| Feb | 0.191 | n.s. | 0.157 | n.s. |
| Feb-Mar | 0.206 | n.s. | 0.229 | n.s. |
| Feb-Apr | 0.162 | n.s. | 0.153 | n.s. |
| Feb-May | 0.104 | n.s. | 0.134 | n.s. |
| Feb-Jun | 0.111 | n.s. | 0.127 | n.s. |
| Feb-Jul | 0.074 | n.s. | 0.118 | n.s. |
| Feb-Aug | 0.078 | n.s. | 0.114 | n.s. |
| Feb-Sep | 0.141 | n.s. | 0.079 | n.s. |
| Feb-Oct | 0.072 | n.s. | 0.048 | n.s. |
| Feb-Nov | -0.021 | n.s. | -0.116 | n.s. |
| Feb-Dec | 0.067 | n.s. | -0.084 | n.s. |
| Feb-JAN | 0.046 | n.s. | -0.043 | n.s. |
| Mar | 0.130 | n.s. | 0.289 | n.s. |
| Mar-Apr | 0.089 | n.s. | 0.093 | n.s. |
| Mar-May | -0.043 | n.s. | 0.008 | n.s. |
| Mar-Jun | -0.024 | n.s. | -0.004 | n.s. |
| Mar-Jul | -0.073 | n.s. | -0.014 | n.s. |
| Mar-Aug | -0.073 | n.s. | -0.025 | n.s. |
| Mar-Sep | 0.015 | n.s. | -0.087 | n.s. |
| Mar-Oct | -0.053 | n.s. | -0.082 | n.s. |
| Mar-Nov | -0.093 | n.s. | -0.270 | n.s. |
| Mar-Dec | 0.013 | n.s. | -0.174 | n.s. |
| Mar-JAN | -0.006 | n.s. | -0.107 | n.s. |
| Mar-FEB | 0.030 | n.s. | -0.070 | n.s. |
| Apr | 0.023 | n.s. | -0.251 | n.s. |
| Apr-May | -0.132 | n.s. | -0.251 | n.s. |
| Apr-Jun | -0.101 | n.s. | -0.235 | n.s. |
| Apr-Jul | -0.138 | n.s. | -0.235 | n.s. |
| Apr-Aug | -0.138 | n.s. | -0.237 | n.s. |
| Apr-Sep | -0.062 | n.s. | -0.270 | n.s. |
| Apr-Oct | -0.103 | n.s. | -0.220 | n.s. |
| Apr-Nov | -0.114 | n.s. | **-0.305** | * |
| Apr-Dec | -0.009 | n.s. | -0.220 | n.s. |
| Apr-JAN | -0.025 | n.s. | -0.156 | n.s. |
| Apr-FEB | 0.011 | n.s. | -0.118 | n.s. |
| Apr-MAR | 0.065 | n.s. | -0.108 | n.s. |
| May | -0.142 | n.s. | -0.138 | n.s. |
| May-Jun | -0.111 | n.s. | -0.122 | n.s. |
| May-Jul | -0.147 | n.s. | -0.136 | n.s. |
| May-Aug | -0.148 | n.s. | -0.140 | n.s. |
| May-Sep | -0.073 | n.s. | -0.190 | n.s. |
| May-Oct | -0.119 | n.s. | -0.165 | n.s. |
| May-Nov | -0.121 | n.s. | -0.257 | n.s. |
| May-Dec | -0.013 | n.s. | -0.169 | n.s. |
| May-JAN | -0.030 | n.s. | -0.112 | n.s. |
| May-FEB | 0.008 | n.s. | -0.080 | n.s. |
| May-MAR | 0.064 | n.s. | -0.073 | n.s. |
| May-APR | 0.052 | n.s. | -0.093 | n.s. |
| Jun | 0.040 | n.s. | -0.044 | n.s. |
| Jun-Jul | -0.090 | n.s. | -0.077 | n.s. |
| Jun-Aug | -0.076 | n.s. | -0.107 | n.s. |
| Jun-Sep | 0.074 | n.s. | -0.185 | n.s. |
| Jun-Oct | -0.028 | n.s. | -0.106 | n.s. |
| Jun-Nov | -0.083 | n.s. | -0.245 | n.s. |
| Jun-Dec | 0.024 | n.s. | -0.154 | n.s. |
| Jun-JAN | 0.004 | n.s. | -0.097 | n.s. |
| Jun-FEB | 0.040 | n.s. | -0.066 | n.s. |
| Jun-MAR | 0.097 | n.s. | -0.059 | n.s. |
| Jun-APR | 0.086 | n.s. | -0.080 | n.s. |
| Jun-MAY | 0.077 | n.s. | -0.093 | n.s. |
| Jul | -0.248 | n.s. | -0.057 | n.s. |
| Jul-Aug | -0.172 | n.s. | -0.119 | n.s. |
| Jul-Sep | 0.061 | n.s. | -0.176 | n.s. |
| Jul-Oct | -0.039 | n.s. | -0.090 | n.s. |
| Jul-Nov | -0.097 | n.s. | -0.247 | n.s. |
| Jul-Dec | 0.022 | n.s. | -0.154 | n.s. |
| Jul-JAN | 0.001 | n.s. | -0.095 | n.s. |
| Jul-FEB | 0.039 | n.s. | -0.064 | n.s. |
| Jul-MAR | 0.100 | n.s. | -0.057 | n.s. |
| Jul-APR | 0.087 | n.s. | -0.079 | n.s. |
| Jul-MAY | 0.078 | n.s. | -0.093 | n.s. |
| Jul-JUN | 0.071 | n.s. | -0.076 | n.s. |
| Aug | 0.010 | n.s. | -0.130 | n.s. |
| Aug-Sep | 0.151 | n.s. | -0.192 | n.s. |
| Aug-Oct | -0.001 | n.s. | -0.090 | n.s. |
| Aug-Nov | -0.076 | n.s. | -0.246 | n.s. |
| Aug-Dec | 0.040 | n.s. | -0.151 | n.s. |
| Aug-JAN | 0.017 | n.s. | -0.093 | n.s. |
| Aug-FEB | 0.054 | n.s. | -0.062 | n.s. |
| Aug-MAR | 0.115 | n.s. | -0.055 | n.s. |
| Aug-APR | 0.102 | n.s. | -0.077 | n.s. |
| Aug-MAY | 0.093 | n.s. | -0.091 | n.s. |
| Aug-JUN | 0.085 | n.s. | -0.074 | n.s. |
| Aug-JUL | 0.091 | n.s. | -0.071 | n.s. |
| Sep | 0.185 | n.s. | -0.171 | n.s. |
| Sep-Oct | -0.003 | n.s. | -0.081 | n.s. |
| Sep-Nov | -0.081 | n.s. | -0.245 | n.s. |
| Sep-Dec | 0.040 | n.s. | -0.151 | n.s. |
| Sep-JAN | 0.017 | n.s. | -0.094 | n.s. |
| Sep-FEB | 0.055 | n.s. | -0.064 | n.s. |
| Sep-MAR | 0.117 | n.s. | -0.058 | n.s. |
| Sep-APR | 0.103 | n.s. | -0.080 | n.s. |
| Sep-MAY | 0.094 | n.s. | -0.095 | n.s. |
| Sep-JUN | 0.086 | n.s. | -0.078 | n.s. |
| Sep-JUL | 0.092 | n.s. | -0.075 | n.s. |
| Sep-AUG | 0.082 | n.s. | -0.075 | n.s. |
| Oct | -0.075 | n.s. | -0.036 | n.s. |
| Oct-Nov | -0.120 | n.s. | -0.227 | n.s. |
| Oct-Dec | 0.010 | n.s. | -0.132 | n.s. |
| Oct-JAN | -0.011 | n.s. | -0.075 | n.s. |
| Oct-FEB | 0.031 | n.s. | -0.045 | n.s. |
| Oct-MAR | 0.094 | n.s. | -0.039 | n.s. |
| Oct-APR | 0.081 | n.s. | -0.061 | n.s. |
| Oct-MAY | 0.071 | n.s. | -0.076 | n.s. |
| Oct-JUN | 0.064 | n.s. | -0.059 | n.s. |
| Oct-JUL | 0.070 | n.s. | -0.056 | n.s. |
| Oct-AUG | 0.061 | n.s. | -0.063 | n.s. |
| Oct-SEP | 0.049 | n.s. | -0.079 | n.s. |
| Nov | -0.096 | n.s. | -0.200 | n.s. |
| Nov-Dec | 0.047 | n.s. | -0.107 | n.s. |
| Nov-JAN | 0.020 | n.s. | -0.059 | n.s. |
| Nov-FEB | 0.059 | n.s. | -0.033 | n.s. |
| Nov-MAR | 0.123 | n.s. | -0.028 | n.s. |
| Nov-APR | 0.109 | n.s. | -0.048 | n.s. |
| Nov-MAY | 0.099 | n.s. | -0.062 | n.s. |
| Nov-JUN | 0.091 | n.s. | -0.047 | n.s. |
| Nov-JUL | 0.097 | n.s. | -0.044 | n.s. |
| Nov-AUG | 0.087 | n.s. | -0.050 | n.s. |
| Nov-SEP | 0.074 | n.s. | -0.064 | n.s. |
| Nov-OCT | -0.022 | n.s. | -0.066 | n.s. |
| Dec | 0.200 | n.s. | 0.009 | n.s. |
| Dec-JAN | 0.127 | n.s. | 0.031 | n.s. |
| Dec-FEB | 0.163 | n.s. | 0.056 | n.s. |
| Dec-MAR | 0.245 | n.s. | 0.057 | n.s. |
| Dec-APR | 0.219 | n.s. | 0.030 | n.s. |
| Dec-MAY | 0.205 | n.s. | 0.007 | n.s. |
| Dec-JUN | 0.190 | n.s. | 0.027 | n.s. |
| Dec-JUL | 0.199 | n.s. | 0.031 | n.s. |
| Dec-AUG | 0.184 | n.s. | 0.026 | n.s. |
| Dec-SEP | 0.165 | n.s. | 0.005 | n.s. |
| Dec-OCT | 0.123 | n.s. | 0.011 | n.s. |
| Dec-NOV | 0.167 | n.s. | 0.083 | n.s. |
| JAN-FEB | 0.071 | n.s. | 0.075 | n.s. |
| JAN-MAR | 0.225 | n.s. | 0.071 | n.s. |
| JAN-APR | 0.186 | n.s. | 0.034 | n.s. |
| JAN-MAY | 0.161 | n.s. | 0.004 | n.s. |
| JAN-JUN | 0.138 | n.s. | 0.031 | n.s. |
| JAN-JUL | 0.152 | n.s. | 0.032 | n.s. |
| JAN-AUG | 0.126 | n.s. | 0.031 | n.s. |
| JAN-SEP | 0.098 | n.s. | 0.003 | n.s. |
| JAN-OCT | 0.063 | n.s. | 0.010 | n.s. |
| JAN-NOV | 0.119 | n.s. | 0.098 | n.s. |
| FEB-MAR | 0.294 | n.s. | 0.061 | n.s. |
| FEB-APR | 0.252 | n.s. | 0.004 | n.s. |
| FEB-MAY | 0.217 | n.s. | -0.049 | n.s. |
| FEB-JUN | 0.194 | n.s. | -0.001 | n.s. |
| FEB-JUL | 0.207 | n.s. | 0.008 | n.s. |
| FEB-AUG | 0.177 | n.s. | 0.007 | n.s. |
| FEB-SEP | 0.143 | n.s. | -0.041 | n.s. |
| FEB-OCT | 0.131 | n.s. | -0.030 | n.s. |
| FEB-NOV | 0.160 | n.s. | 0.108 | n.s. |
| MAR-APR | 0.269 | n.s. | -0.096 | n.s. |
| MAR-MAY | 0.194 | n.s. | -0.217 | n.s. |
| MAR-JUN | 0.160 | n.s. | -0.125 | n.s. |
| MAR-JUL | 0.177 | n.s. | -0.110 | n.s. |
| MAR-AUG | 0.133 | n.s. | -0.114 | n.s. |
| MAR-SEP | 0.085 | n.s. | -0.172 | n.s. |
| MAR-OCT | 0.120 | n.s. | -0.125 | n.s. |
| MAR-NOV | 0.170 | n.s. | 0.091 | n.s. |
| APR-MAY | -0.124 | n.s. | -0.284 | n.s. |
| APR-JUN | -0.132 | n.s. | -0.159 | n.s. |
| APR-JUL | -0.095 | n.s. | -0.138 | n.s. |
| APR-AUG | -0.144 | n.s. | -0.140 | n.s. |
| APR-SEP | -0.175 | n.s. | -0.203 | n.s. |
| APR-OCT | -0.033 | n.s. | -0.195 | n.s. |
| APR-NOV | 0.045 | n.s. | 0.095 | n.s. |
| MAY-JUN | -0.097 | n.s. | -0.015 | n.s. |
| MAY-JUL | -0.049 | n.s. | 0.013 | n.s. |
| MAY-AUG | -0.132 | n.s. | 0.009 | n.s. |
| MAY-SEP | -0.167 | n.s. | -0.109 | n.s. |
| MAY-OCT | 0.025 | n.s. | -0.070 | n.s. |
| MAY-NOV | 0.093 | n.s. | 0.174 | n.s. |
| JUN-JUL | 0.017 | n.s. | **0.364** | * |
| JUN-AUG | -0.141 | n.s. | **0.350** | * |
| JUN-SEP | -0.162 | n.s. | 0.028 | n.s. |
| JUN-OCT | 0.046 | n.s. | 0.032 | n.s. |
| JUN-NOV | 0.108 | n.s. | 0.213 | n.s. |
| JUL-AUG | -0.091 | n.s. | 0.188 | n.s. |
| JUL-SEP | -0.133 | n.s. | -0.146 | n.s. |
| JUL-OCT | 0.134 | n.s. | -0.047 | n.s. |
| JUL-NOV | 0.220 | n.s. | 0.159 | n.s. |
| AUG-SEP | -0.193 | n.s. | -0.173 | n.s. |
| AUG-OCT | 0.037 | n.s. | -0.062 | n.s. |
| AUG-NOV | 0.093 | n.s. | 0.146 | n.s. |
| SEP-OCT | 0.150 | n.s. | -0.059 | n.s. |
| SEP-NOV | 0.111 | n.s. | 0.148 | n.s. |
| OCT-NOV | 0.139 | n.s. | 0.213 | n.s. |
