# Supplementary material for: Beyond width and density: stable carbon and oxygen isotopes in cork-rings provide insights of physiological responses to water stress in Quercus suber L
Source: PeerJ. 2022 Nov 14;10:e14270. doi: 10.7717/peerj.14270 (PMC9671033; doi:10.7717/peerj.14270)
Supplement: Table S3 [file peerj-10-14270-s003.zip › Table S3_Supplementary_Material_Correlations_Climate_OxygenIsotope.docx]

Table S3: A) Study areas Benavente (CL) and Grândola (BS). Pearson’s correlations coefficient ( r ) between Mean Temperature and cork-ring’s δ^18^O. p-value<0.01 (**); p-value < 0.05 (*); p-value> 0.05 (non-significant at alpha level of 0.05, n.s.)

| Period (months) | Benavente - CL (n= 34) | | Grândola - BS (n=44) | |
| --- | --- | --- | --- | --- |
|  | Pearson's Correlation coefficient ( r) | p-value | Pearson's Correlation coefficient ( r) | p-value |
| Feb | -0.104 | n.s. | -0.144 | n.s. |
| Mar | -0.079 | n.s. | 0.006 | n.s. |
| Apr | -0.092 | n.s. | 0.132 | n.s. |
| May | **-0.345** | ***** | -0.219 | n.s. |
| Jun | -0.231 | n.s. | -0.104 | n.s. |
| Jul | -0.218 | n.s. | -0.207 | n.s. |
| Aug | -0.326 | n.s. | -0.206 | n.s. |
| Sep | -0.164 | n.s. | 0.010 | n.s. |
| Oct | -0.178 | n.s. | 0.085 | n.s. |
| Nov | **-0.251** | ***** | -0.103 | n.s. |
| Dec | -0.146 | n.s. | -0.108 | **n.s.** |
| JAN | -0.091 | n.s. | -0.143 | n.s. |
| FEB | 0.070 | n.s. | 0.028 | n.s. |
| MAR | -0.084 | n.s. | -0.101 | n.s. |
| APR | -0.077 | n.s. | -0.097 | n.s. |
| MAY | -0.150 | n.s. | -0.068 | n.s. |
| JUN | 0.193 | n.s. | -0.100 | n.s. |
| JUL | 0.065 | n.s. | -0.046 | n.s. |
| AUG | 0.169 | n.s. | -0.101 | n.s. |
| SEP | -0.171 | n.s. | -0.138 | n.s. |
| OCT | 0.078 | n.s. | 0.105 | n.s. |
| NOV | -0.124 | n.s. | -0.133 | n.s. |
| Feb | -0.104 | n.s. | -0.144 | n.s. |
| Feb-Mar | -0.096 | n.s. | -0.065 | n.s. |
| Feb-Apr | -0.101 | n.s. | 0.004 | n.s. |
| Feb-May | -0.163 | n.s. | -0.064 | n.s. |
| Feb-Jun | -0.231 | n.s. | -0.093 | n.s. |
| Feb-Jul | -0.242 | n.s. | -0.127 | n.s. |
| Feb-Aug | -0.268 | n.s. | -0.149 | n.s. |
| Feb-Sep | -0.260 | n.s. | -0.143 | n.s. |
| Feb-Oct | -0.272 | n.s. | -0.116 | n.s. |
| Feb-Nov | -0.235 | n.s. | -0.118 | n.s. |
| Feb-Dec | -0.230 | n.s. | -0.122 | n.s. |
| Feb-JAN | -0.223 | n.s. | -0.131 | n.s. |
| Mar | -0.079 | n.s. | 0.006 | n.s. |
| Mar-Apr | -0.092 | n.s. | 0.070 | n.s. |
| Mar-May | -0.172 | n.s. | -0.028 | n.s. |
| Mar-Jun | -0.256 | n.s. | -0.064 | n.s. |
| Mar-Jul | -0.266 | n.s. | -0.109 | n.s. |
| Mar-Aug | -0.290 | n.s. | -0.136 | n.s. |
| Mar-Sep | -0.278 | n.s. | -0.129 | n.s. |
| Mar-Oct | -0.287 | n.s. | -0.101 | n.s. |
| Mar-Nov | -0.238 | n.s. | -0.106 | n.s. |
| Mar-Dec | -0.233 | n.s. | -0.113 | n.s. |
| Mar-JAN | -0.225 | n.s. | -0.123 | n.s. |
| Mar-FEB | -0.192 | n.s. | -0.107 | n.s. |
| Apr | -0.092 | n.s. | 0.132 | n.s. |
| Apr-May | -0.226 | **n.s.** | -0.049 | n.s. |
| Apr-Jun | -0.337 | **n.s.** | -0.098 | n.s. |
| Apr-Jul | -0.332 | n.s. | -0.154 | n.s. |
| Apr-Aug | **-0.346** | ***** | -0.176 | n.s. |
| Apr-Sep | -0.322 | n.s. | -0.164 | n.s. |
| Apr-Oct | -0.329 | n.s. | -0.130 | n.s. |
| Apr-Nov | -0.264 | n.s. | -0.129 | n.s. |
| Apr-Dec | -0.256 | n.s. | -0.133 | n.s. |
| Apr-JAN | -0.244 | n.s. | -0.143 | n.s. |
| Apr-FEB | -0.205 | n.s. | -0.123 | n.s. |
| Apr-MAR | -0.197 | n.s. | -0.125 | n.s. |
| May | **-0.345** | ***** | -0.219 | n.s. |
| May-Jun | **-0.373** | ***** | -0.222 | n.s. |
| May-Jul | **-0.350** | ***** | -0.257 | n.s. |
| May-Aug | **-0.359** | ***** | -0.250 | n.s. |
| May-Sep | -0.337 | n.s. | -0.228 | n.s. |
| May-Oct | **-0.344** | ***** | -0.201 | n.s. |
| May-Nov | -0.268 | n.s. | -0.183 | n.s. |
| May-Dec | -0.261 | n.s. | -0.179 | n.s. |
| May-JAN | -0.248 | n.s. | -0.185 | n.s. |
| May-FEB | -0.207 | n.s. | -0.159 | n.s. |
| May-MAR | -0.200 | n.s. | -0.159 | n.s. |
| May-APR | -0.193 | n.s. | -0.161 | n.s. |
| Jun | -0.231 | n.s. | -0.104 | n.s. |
| Jun-Jul | -0.272 | n.s. | -0.185 | n.s. |
| Jun-Aug | -0.312 | n.s. | -0.210 | n.s. |
| Jun-Sep | -0.296 | n.s. | -0.185 | n.s. |
| Jun-Oct | -0.305 | n.s. | -0.149 | n.s. |
| Jun-Nov | -0.218 | n.s. | -0.146 | n.s. |
| Jun-Dec | -0.222 | n.s. | -0.145 | n.s. |
| Jun-JAN | -0.214 | n.s. | -0.157 | n.s. |
| Jun-FEB | -0.175 | n.s. | -0.130 | n.s. |
| Jun-MAR | -0.171 | n.s. | -0.132 | n.s. |
| Jun-APR | -0.167 | n.s. | -0.136 | n.s. |
| Jun-MAY | -0.170 | n.s. | -0.137 | n.s. |
| Jul | -0.218 | n.s. | -0.207 | n.s. |
| Jul-Aug | -0.296 | n.s. | -0.227 | n.s. |
| Jul-Sep | -0.264 | n.s. | -0.180 | n.s. |
| Jul-Oct | -0.277 | n.s. | -0.120 | n.s. |
| Jul-Nov | -0.165 | n.s. | -0.123 | n.s. |
| Jul-Dec | -0.186 | n.s. | -0.127 | n.s. |
| Jul-JAN | -0.183 | n.s. | -0.141 | n.s. |
| Jul-FEB | -0.146 | n.s. | -0.114 | n.s. |
| Jul-MAR | -0.145 | n.s. | -0.118 | n.s. |
| Jul-APR | -0.143 | n.s. | -0.124 | n.s. |
| Jul-MAY | -0.147 | n.s. | -0.126 | n.s. |
| Jul-JUN | -0.118 | n.s. | -0.131 | n.s. |
| Aug | -0.326 | n.s. | -0.206 | n.s. |
| Aug-Sep | -0.258 | n.s. | -0.130 | n.s. |
| Aug-Oct | -0.263 | n.s. | -0.052 | n.s. |
| Aug-Nov | -0.136 | n.s. | -0.085 | n.s. |
| Aug-Dec | -0.170 | n.s. | -0.102 | n.s. |
| Aug-JAN | -0.168 | n.s. | -0.120 | n.s. |
| Aug-FEB | -0.130 | n.s. | -0.093 | n.s. |
| Aug-MAR | -0.131 | n.s. | -0.099 | n.s. |
| Aug-APR | -0.130 | n.s. | -0.107 | n.s. |
| Aug-MAY | -0.135 | n.s. | -0.111 | n.s. |
| Aug-JUN | -0.104 | n.s. | -0.119 | n.s. |
| Aug-JUL | -0.085 | n.s. | -0.124 | n.s. |
| Sep | -0.164 | n.s. | 0.010 | n.s. |
| Sep-Oct | -0.192 | n.s. | 0.086 | n.s. |
| Sep-Nov | -0.065 | n.s. | -0.020 | n.s. |
| Sep-Dec | -0.129 | n.s. | -0.061 | n.s. |
| Sep-JAN | -0.138 | n.s. | -0.090 | n.s. |
| Sep-FEB | -0.102 | n.s. | -0.065 | n.s. |
| Sep-MAR | -0.105 | n.s. | -0.075 | n.s. |
| Sep-APR | -0.106 | n.s. | -0.085 | n.s. |
| Sep-MAY | -0.111 | n.s. | -0.092 | n.s. |
| Sep-JUN | -0.080 | n.s. | -0.102 | n.s. |
| Sep-JUL | -0.061 | n.s. | -0.106 | n.s. |
| Sep-AUG | -0.037 | n.s. | -0.112 | n.s. |
| Oct | -0.178 | n.s. | 0.085 | n.s. |
| Oct-Nov | -0.033 | n.s. | -0.024 | n.s. |
| Oct-Dec | -0.137 | n.s. | -0.064 | n.s. |
| Oct-JAN | -0.141 | n.s. | -0.090 | n.s. |
| Oct-FEB | -0.099 | n.s. | -0.066 | n.s. |
| Oct-MAR | -0.101 | n.s. | -0.076 | n.s. |
| Oct-APR | -0.101 | n.s. | -0.087 | n.s. |
| Oct-MAY | -0.106 | n.s. | -0.092 | n.s. |
| Oct-JUN | -0.070 | n.s. | -0.102 | n.s. |
| Oct-JUL | -0.050 | n.s. | -0.105 | n.s. |
| Oct-AUG | -0.024 | n.s. | -0.112 | n.s. |
| Oct-SEP | -0.032 | n.s. | -0.122 | n.s. |
| Nov | -0.251 | n.s. | -0.103 | n.s. |
| Nov-Dec | -0.168 | n.s. | -0.117 | n.s. |
| Nov-JAN | -0.148 | n.s. | -0.133 | n.s. |
| Nov-FEB | -0.090 | n.s. | -0.096 | n.s. |
| Nov-MAR | -0.089 | n.s. | -0.103 | n.s. |
| Nov-APR | -0.088 | n.s. | -0.112 | n.s. |
| Nov-MAY | -0.092 | n.s. | -0.116 | n.s. |
| Nov-JUN | -0.052 | n.s. | -0.123 | n.s. |
| Nov-JUL | -0.030 | n.s. | -0.129 | n.s. |
| Nov-AUG | -0.003 | n.s. | -0.134 | n.s. |
| Nov-SEP | -0.010 | n.s. | -0.142 | n.s. |
| Nov-OCT | -0.085 | n.s. | -0.129 | n.s. |
| Dec | -0.146 | n.s. | -0.108 | n.s. |
| Dec-JAN | -0.125 | n.s. | -0.136 | n.s. |
| Dec-FEB | 0.170 | n.s. | 0.033 | n.s. |
| Dec-MAR | -0.065 | n.s. | -0.095 | n.s. |
| Dec-APR | -0.072 | n.s. | -0.106 | n.s. |
| Dec-MAY | -0.088 | n.s. | -0.111 | n.s. |
| Dec-JUN | -0.048 | n.s. | -0.121 | n.s. |
| Dec-JUL | -0.030 | n.s. | -0.128 | n.s. |
| Dec-AUG | -0.005 | n.s. | -0.132 | n.s. |
| Dec-SEP | -0.019 | n.s. | -0.141 | n.s. |
| Dec-OCT | -0.095 | n.s. | -0.127 | n.s. |
| Dec-NOV | -0.102 | n.s. | -0.131 | n.s. |
| JAN-FEB | -0.013 | n.s. | -0.059 | n.s. |
| JAN-MAR | -0.034 | n.s. | -0.080 | n.s. |
| JAN-APR | -0.048 | n.s. | -0.099 | n.s. |
| JAN-MAY | -0.070 | n.s. | -0.108 | n.s. |
| JAN-JUN | -0.023 | n.s. | -0.122 | n.s. |
| JAN-JUL | -0.005 | n.s. | -0.128 | n.s. |
| JAN-AUG | 0.020 | n.s. | -0.133 | n.s. |
| JAN-SEP | 0.005 | n.s. | -0.142 | n.s. |
| JAN-OCT | -0.071 | n.s. | -0.126 | n.s. |
| JAN-NOV | -0.080 | n.s. | -0.130 | n.s. |
| FEB-MAR | 0.007 | n.s. | -0.027 | n.s. |
| FEB-APR | -0.021 | n.s. | -0.062 | n.s. |
| FEB-MAY | -0.054 | n.s. | -0.077 | n.s. |
| FEB-JUN | 0.006 | n.s. | -0.099 | n.s. |
| FEB-JUL | 0.022 | n.s. | -0.106 | n.s. |
| FEB-AUG | 0.048 | n.s. | -0.112 | n.s. |
| FEB-SEP | 0.029 | n.s. | -0.125 | n.s. |
| FEB-OCT | -0.034 | n.s. | -0.108 | n.s. |
| FEB-NOV | -0.049 | n.s. | -0.115 | n.s. |
| MAR-APR | -0.089 | n.s. | -0.127 | n.s. |
| MAR-MAY | -0.120 | n.s. | -0.118 | n.s. |
| MAR-JUN | -0.026 | n.s. | -0.128 | n.s. |
| MAR-JUL | 0.002 | n.s. | -0.120 | n.s. |
| MAR-AUG | 0.037 | n.s. | -0.125 | n.s. |
| MAR-SEP | 0.014 | n.s. | -0.138 | n.s. |
| MAR-OCT | -0.024 | n.s. | -0.118 | n.s. |
| MAR-NOV | -0.043 | n.s. | -0.126 | n.s. |
| APR-MAY | -0.123 | n.s. | -0.101 | n.s. |
| APR-JUN | 0.004 | n.s. | -0.121 | n.s. |
| APR-JUL | 0.031 | n.s. | -0.120 | n.s. |
| APR-AUG | 0.066 | n.s. | -0.127 | n.s. |
| APR-SEP | 0.039 | n.s. | -0.140 | n.s. |
| APR-OCT | -0.012 | n.s. | -0.118 | n.s. |
| APR-NOV | -0.036 | n.s. | -0.127 | n.s. |
| MAY-JUN | 0.055 | n.s. | -0.105 | n.s. |
| MAY-JUL | 0.069 | n.s. | -0.087 | n.s. |
| MAY-AUG | 0.102 | n.s. | -0.100 | n.s. |
| MAY-SEP | 0.070 | n.s. | -0.118 | n.s. |
| MAY-OCT | 0.032 | n.s. | -0.095 | n.s. |
| MAY-NOV | -0.003 | n.s. | -0.108 | n.s. |
| JUN-JUL | 0.146 | n.s. | -0.094 | n.s. |
| JUN-AUG | 0.162 | n.s. | -0.108 | n.s. |
| JUN-SEP | 0.123 | n.s. | -0.128 | n.s. |
| JUN-OCT | 0.072 | n.s. | -0.100 | n.s. |
| JUN-NOV | 0.025 | n.s. | -0.115 | n.s. |
| JUL-AUG | 0.117 | n.s. | -0.085 | n.s. |
| JUL-SEP | 0.067 | n.s. | -0.115 | n.s. |
| JUL-OCT | 0.010 | n.s. | -0.081 | n.s. |
| JUL-NOV | -0.031 | n.s. | -0.099 | n.s. |
| AUG-SEP | 0.053 | n.s. | -0.131 | n.s. |
| AUG-OCT | 0.038 | n.s. | -0.088 | n.s. |
| AUG-NOV | -0.027 | n.s. | -0.113 | n.s. |
| SEP-OCT | -0.022 | n.s. | -0.056 | n.s. |
| SEP-NOV | -0.083 | n.s. | -0.096 | n.s. |
| OCT-NOV | -0.032 | n.s. | -0.003 | n.s. |

Table S3: B) Study areas Benavente (CL) and Grândola (BS). Pearson’s correlations coefficient ( r ) between Precipitation and cork-ring’s δ^18^O. p-value<0.01 (**); p-value < 0.05 (*); p-value> 0.05 (non-significant at alpha level of 0.05, n.s.)

| Period (months) | Benavente - CL (n= 34) | | Grândola - BS (n=44) | |
| --- | --- | --- | --- | --- |
|  | Pearson's Correlation coefficient ( r) | p-value | Pearson's Correlation coefficient ( r) | p-value |
| Feb | -0.085 | n.s. | -0.096 | n.s. |
| Mar | 0.028 | n.s. | -0.035 | n.s. |
| Apr | -0.063 | n.s. | -0.180 | n.s. |
| May | 0.020 | n.s. | 0.169 | n.s. |
| Jun | 0.318 | n.s. | 0.171 | n.s. |
| Jul | -0.037 | n.s. | -0.099 | n.s. |
| Aug | 0.087 | n.s. | **0.362** | ***** |
| Sep | -0.174 | n.s. | 0.018 | n.s. |
| Oct | 0.119 | n.s. | -0.078 | n.s. |
| Nov | 0.205 | n.s. | 0.155 | n.s. |
| Dec | 0.222 | n.s. | 0.042 | n.s. |
| JAN | -0.218 | n.s. | -0.065 | n.s. |
| FEB | 0.078 | n.s. | 0.106 | n.s. |
| MAR | **0.411** | ***** | -0.240 | n.s. |
| APR | 0.085 | n.s. | -0.069 | n.s. |
| MAY | **0.411** | ***** | 0.071 | n.s. |
| JUN | -0.114 | n.s. | 0.267 | n.s. |
| JUL | 0.045 | n.s. | 0.082 | n.s. |
| AUG | -0.050 | n.s. | -0.093 | n.s. |
| SEP | -0.118 | n.s. | -0.088 | n.s. |
| OCT | 0.074 | n.s. | -0.077 | n.s. |
| NOV | 0.006 | n.s. | 0.148 | n.s. |
| Feb | -0.085 | n.s. | -0.096 | n.s. |
| Feb-Mar | -0.052 | n.s. | -0.083 | n.s. |
| Feb-Apr | -0.063 | n.s. | -0.142 | n.s. |
| Feb-May | -0.062 | n.s. | -0.112 | n.s. |
| Feb-Jun | 0.010 | n.s. | -0.088 | n.s. |
| Feb-Jul | 0.005 | n.s. | -0.093 | n.s. |
| Feb-Aug | 0.016 | n.s. | -0.078 | n.s. |
| Feb-Sep | -0.040 | n.s. | -0.076 | n.s. |
| Feb-Oct | 0.057 | n.s. | -0.100 | n.s. |
| Feb-Nov | 0.175 | n.s. | 0.019 | n.s. |
| Feb-Dec | 0.216 | n.s. | 0.041 | n.s. |
| Feb-JAN | 0.172 | n.s. | -0.004 | n.s. |
| Mar | 0.028 | n.s. | -0.035 | n.s. |
| Mar-Apr | -0.025 | n.s. | -0.171 | n.s. |
| Mar-May | -0.009 | n.s. | -0.071 | n.s. |
| Mar-Jun | 0.102 | n.s. | -0.020 | n.s. |
| Mar-Jul | 0.088 | n.s. | -0.036 | n.s. |
| Mar-Aug | 0.107 | n.s. | -0.003 | n.s. |
| Mar-Sep | 0.025 | n.s. | -0.003 | n.s. |
| Mar-Oct | 0.117 | n.s. | -0.060 | n.s. |
| Mar-Nov | 0.201 | n.s. | 0.104 | n.s. |
| Mar-Dec | 0.235 | n.s. | 0.096 | n.s. |
| Mar-JAN | 0.190 | n.s. | 0.037 | n.s. |
| Mar-FEB | 0.185 | n.s. | 0.064 | n.s. |
| Apr | -0.063 | n.s. | -0.180 | n.s. |
| Apr-May | -0.028 | n.s. | -0.029 | n.s. |
| Apr-Jun | 0.083 | n.s. | 0.012 | n.s. |
| Apr-Jul | 0.067 | n.s. | -0.002 | n.s. |
| Apr-Aug | 0.084 | n.s. | 0.024 | n.s. |
| Apr-Sep | 0.009 | n.s. | 0.023 | n.s. |
| Apr-Oct | 0.104 | n.s. | -0.035 | n.s. |
| Apr-Nov | 0.181 | n.s. | 0.090 | n.s. |
| Apr-Dec | 0.222 | n.s. | 0.088 | n.s. |
| Apr-JAN | 0.178 | n.s. | 0.040 | n.s. |
| Apr-FEB | 0.175 | n.s. | 0.064 | n.s. |
| Apr-MAR | 0.224 | n.s. | 0.017 | n.s. |
| May | 0.020 | n.s. | 0.169 | n.s. |
| May-Jun | 0.119 | n.s. | 0.188 | n.s. |
| May-Jul | 0.102 | n.s. | 0.170 | n.s. |
| May-Aug | 0.119 | n.s. | 0.197 | n.s. |
| May-Sep | 0.048 | n.s. | 0.169 | n.s. |
| May-Oct | 0.139 | n.s. | 0.048 | n.s. |
| May-Nov | 0.198 | n.s. | 0.153 | n.s. |
| May-Dec | 0.241 | n.s. | 0.126 | n.s. |
| May-JAN | 0.194 | n.s. | 0.068 | n.s. |
| May-FEB | 0.190 | n.s. | 0.089 | n.s. |
| May-MAR | 0.240 | n.s. | 0.042 | n.s. |
| May-APR | 0.248 | n.s. | 0.036 | n.s. |
| Jun | 0.318 | n.s. | 0.171 | n.s. |
| Jun-Jul | 0.227 | n.s. | 0.103 | n.s. |
| Jun-Aug | 0.236 | n.s. | 0.200 | n.s. |
| Jun-Sep | 0.047 | n.s. | 0.114 | n.s. |
| Jun-Oct | 0.123 | n.s. | -0.026 | n.s. |
| Jun-Nov | 0.214 | n.s. | 0.122 | n.s. |
| Jun-Dec | 0.252 | n.s. | 0.103 | n.s. |
| Jun-JAN | 0.202 | n.s. | 0.048 | n.s. |
| Jun-FEB | 0.197 | n.s. | 0.072 | n.s. |
| Jun-MAR | 0.249 | n.s. | 0.024 | n.s. |
| Jun-APR | 0.258 | n.s. | 0.018 | n.s. |
| Jun-MAY | 0.283 | n.s. | 0.025 | n.s. |
| Jul | -0.037 | n.s. | -0.099 | n.s. |
| Jul-Aug | 0.033 | n.s. | 0.093 | n.s. |
| Jul-Sep | -0.119 | n.s. | 0.033 | n.s. |
| Jul-Oct | 0.053 | n.s. | -0.054 | n.s. |
| Jul-Nov | 0.187 | n.s. | 0.105 | n.s. |
| Jul-Dec | 0.237 | n.s. | 0.092 | n.s. |
| Jul-JAN | 0.185 | n.s. | 0.038 | n.s. |
| Jul-FEB | 0.181 | n.s. | 0.064 | n.s. |
| Jul-MAR | 0.237 | n.s. | 0.015 | n.s. |
| Jul-APR | 0.247 | n.s. | 0.009 | n.s. |
| Jul-MAY | 0.274 | n.s. | 0.017 | n.s. |
| Jul-JUN | 0.258 | n.s. | 0.030 | n.s. |
| Aug | 0.087 | n.s. | **0.362** | ***** |
| Aug-Sep | -0.110 | n.s. | 0.081 | n.s. |
| Aug-Oct | 0.058 | n.s. | -0.046 | n.s. |
| Aug-Nov | 0.197 | n.s. | 0.114 | n.s. |
| Aug-Dec | 0.246 | n.s. | 0.097 | n.s. |
| Aug-JAN | 0.192 | n.s. | 0.042 | n.s. |
| Aug-FEB | 0.188 | n.s. | 0.067 | n.s. |
| Aug-MAR | 0.243 | n.s. | 0.018 | n.s. |
| Aug-APR | 0.254 | n.s. | 0.013 | n.s. |
| Aug-MAY | 0.282 | n.s. | 0.020 | n.s. |
| Aug-JUN | 0.264 | n.s. | 0.033 | n.s. |
| Aug-JUL | 0.266 | n.s. | 0.035 | n.s. |
| Sep | -0.174 | n.s. | 0.018 | n.s. |
| Sep-Oct | 0.050 | n.s. | -0.075 | n.s. |
| Sep-Nov | 0.200 | n.s. | 0.091 | n.s. |
| Sep-Dec | 0.248 | n.s. | 0.071 | n.s. |
| Sep-JAN | 0.192 | n.s. | 0.012 | n.s. |
| Sep-FEB | 0.187 | n.s. | 0.032 | n.s. |
| Sep-MAR | 0.243 | n.s. | -0.021 | n.s. |
| Sep-APR | 0.253 | n.s. | -0.027 | n.s. |
| Sep-MAY | 0.282 | n.s. | -0.019 | n.s. |
| Sep-JUN | 0.264 | n.s. | -0.006 | n.s. |
| Sep-JUL | 0.266 | n.s. | -0.005 | n.s. |
| Sep-AUG | 0.265 | n.s. | -0.006 | n.s. |
| Oct | 0.119 | n.s. | -0.078 | n.s. |
| Oct-Nov | 0.232 | n.s. | 0.107 | n.s. |
| Oct-Dec | 0.278 | n.s. | 0.090 | n.s. |
| Oct-JAN | 0.220 | n.s. | 0.034 | n.s. |
| Oct-FEB | 0.211 | n.s. | 0.059 | n.s. |
| Oct-MAR | 0.265 | n.s. | 0.012 | n.s. |
| Oct-APR | 0.275 | n.s. | 0.006 | n.s. |
| Oct-MAY | 0.303 | n.s. | 0.014 | n.s. |
| Oct-JUN | 0.285 | n.s. | 0.027 | n.s. |
| Oct-JUL | 0.288 | n.s. | 0.028 | n.s. |
| Oct-AUG | 0.287 | n.s. | -0.007 | n.s. |
| Oct-SEP | 0.270 | n.s. | -0.016 | n.s. |
| Nov | 0.205 | n.s. | 0.155 | n.s. |
| Nov-Dec | 0.257 | n.s. | 0.109 | n.s. |
| Nov-JAN | 0.188 | n.s. | 0.051 | n.s. |
| Nov-FEB | 0.178 | n.s. | 0.072 | n.s. |
| Nov-MAR | 0.237 | n.s. | 0.028 | n.s. |
| Nov-APR | 0.247 | n.s. | 0.023 | n.s. |
| Nov-MAY | 0.277 | n.s. | 0.029 | n.s. |
| Nov-JUN | 0.259 | n.s. | 0.042 | n.s. |
| Nov-JUL | 0.262 | n.s. | 0.043 | n.s. |
| Nov-AUG | 0.261 | n.s. | 0.013 | n.s. |
| Nov-SEP | 0.242 | n.s. | 0.005 | n.s. |
| Nov-OCT | 0.224 | n.s. | -0.009 | n.s. |
| Dec | 0.222 | n.s. | 0.042 | n.s. |
| Dec-JAN | 0.113 | n.s. | -0.017 | n.s. |
| Dec-FEB | 0.118 | n.s. | 0.021 | n.s. |
| Dec-MAR | 0.208 | n.s. | -0.035 | n.s. |
| Dec-APR | 0.218 | n.s. | -0.042 | n.s. |
| Dec-MAY | 0.268 | n.s. | -0.030 | n.s. |
| Dec-JUN | 0.246 | n.s. | -0.014 | n.s. |
| Dec-JUL | 0.248 | n.s. | -0.012 | n.s. |
| Dec-AUG | 0.245 | n.s. | -0.051 | n.s. |
| Dec-SEP | 0.222 | n.s. | -0.062 | n.s. |
| Dec-OCT | 0.215 | n.s. | -0.087 | n.s. |
| Dec-NOV | 0.208 | n.s. | -0.041 | n.s. |
| JAN-FEB | -.0458 | n.s. | .0011 | n.s. |
| JAN-MAR | .1396 | n.s. | -.0723 | n.s. |
| JAN-APR | .1639 | n.s. | -.0806 | n.s. |
| JAN-MAY | .2453 | n.s. | -.0661 | n.s. |
| JAN-JUN | .2088 | n.s. | -.0427 | n.s. |
| JAN-JUL | .2120 | n.s. | -.0825 | n.s. |
| JAN-AUG | .2056 | n.s. | -.0838 | n.s. |
| JAN-SEP | .1714 | n.s. | -.0975 | n.s. |
| JAN-OCT | .1752 | n.s. | -.1278 | n.s. |
| JAN-NOV | .1448 | n.s. | -.0626 | n.s. |
| FEB-MAR | 0.243 | n.s. | -0.077 | n.s. |
| FEB-APR | 0.268 | n.s. | -0.092 | n.s. |
| FEB-MAY | **0.346** | ***** | -0.084 | n.s. |
| FEB-JUN | 0.308 | n.s. | -0.045 | n.s. |
| FEB-JUL | 0.309 | n.s. | -0.043 | n.s. |
| FEB-AUG | 0.297 | n.s. | -0.045 | n.s. |
| FEB-SEP | 0.255 | n.s. | -0.070 | n.s. |
| FEB-OCT | 0.264 | n.s. | -0.107 | n.s. |
| FEB-NOV | 0.211 | n.s. | -0.014 | n.s. |
| MAR-APR | **0.404** | ***** | -0.287 | n.s. |
| MAR-MAY | **0.497** | ****** | -0.285 | n.s. |
| MAR-JUN | **0.428** | ***** | -0.220 | n.s. |
| MAR-JUL | **0.416** | ***** | -0.225 | n.s. |
| MAR-AUG | **0.399** | ***** | -0.230 | n.s. |
| MAR-SEP | 0.332 | n.s. | -0.220 | n.s. |
| MAR-OCT | 0.321 | n.s. | -0.237 | n.s. |
| MAR-NOV | 0.276 | n.s. | -0.088 | n.s. |
| APR-MAY | 0.281 | n.s. | -0.001 | n.s. |
| APR-JUN | 0.199 | n.s. | 0.087 | n.s. |
| APR-JUL | 0.196 | n.s. | 0.095 | n.s. |
| APR-AUG | 0.182 | n.s. | 0.090 | n.s. |
| APR-SEP | 0.104 | n.s. | 0.025 | n.s. |
| APR-OCT | 0.168 | n.s. | -0.054 | n.s. |
| APR-NOV | 0.150 | n.s. | 0.100 | n.s. |
| MAY-JUN | 0.213 | n.s. | 0.166 | n.s. |
| MAY-JUL | 0.219 | n.s. | 0.169 | n.s. |
| MAY-AUG | 0.206 | n.s. | 0.165 | n.s. |
| MAY-SEP | 0.080 | n.s. | 0.075 | n.s. |
| MAY-OCT | 0.147 | n.s. | -0.018 | n.s. |
| MAY-NOV | 0.125 | n.s. | 0.118 | n.s. |
| JUN-JUL | -0.092 | n.s. | 0.256 | n.s. |
| JUN-AUG | -0.132 | n.s. | 0.243 | n.s. |
| JUN-SEP | -0.171 | n.s. | 0.051 | n.s. |
| JUN-OCT | 0.016 | n.s. | -0.048 | n.s. |
| JUN-NOV | 0.017 | n.s. | 0.080 | n.s. |
| JUL-AUG | -0.014 | n.s. | 0.030 | n.s. |
| JUL-SEP | -0.115 | n.s. | -0.087 | n.s. |
| JUL-OCT | 0.111 | n.s. | -0.102 | n.s. |
| JUL-NOV | 0.142 | n.s. | 0.037 | n.s. |
| AUG-SEP | -0.133 | n.s. | -0.093 | n.s. |
| AUG-OCT | 0.037 | n.s. | -0.106 | n.s. |
| AUG-NOV | 0.032 | n.s. | 0.033 | n.s. |
| SEP-OCT | 0.115 | n.s. | -0.103 | n.s. |
| SEP-NOV | 0.026 | n.s. | 0.035 | n.s. |
| OCT-NOV | 0.061 | n.s. | 0.067 | n.s. |
